# Causal relationships of gut microbiota, plasma metabolites, and metabolite ratios with diffuse large B-cell lymphoma: a Mendelian randomization study

**DOI:** 10.3389/fmicb.2024.1356437

**Published:** 2024-05-27

**Authors:** Jingrong Qian, Wen Zheng, Jun Fang, Shiliang Cheng, Yanli Zhang, Xuewei Zhuang, Chao Song

**Affiliations:** ^1^Department of Clinical Laboratory, Shandong Provincial Third Hospital, Shandong University, Jinan, Shandong, China; ^2^Department of Medical Engineering, Shandong Provincial Third Hospital, Shandong University, Jinan, Shandong, China; ^3^Department of Administration, Shandong Provincial Third Hospital, Shandong University, Jinan, Shandong, China

**Keywords:** gut microbiota, plasma metabolites, metabolite ratios, DLBCL, Mendelian randomization

## Abstract

**Background:**

Recent studies have revealed changes in microbiota constitution and metabolites associated with tumor progression, however, no causal relation between microbiota or metabolites and diffuse large B-cell lymphoma (DLBCL) has yet been reported.

**Methods:**

We download a microbiota dataset from the MiBioGen study, a metabolites dataset from the Canadian Longitudinal Study on Aging (CLSA) study, and a DLBCL dataset from Integrative Epidemiology Unit Open genome-wide association study (GWAS) project. Mendelian randomization (MR) analysis was conducted using the R packages, TwoSampleMR and MR-PRESSO. Five MR methods were used: MR-Egger, inverse variance weighting (IVW), weighted median, simple mode, and weighted mode. Reverse MR analyses were also conducted to explore the causal effects of DLBCL on the microbiome, metabolites, and metabolite ratios. Pleiotropy was evaluated by MR Egger regression and MR-PRESSO global analyses, heterogeneity was assessed by Cochran’s Q-test, and stability analyzed using the leave-one-out method.

**Results:**

119 microorganisms, 1,091 plasma metabolite, and 309 metabolite ratios were analyzed. According to IVW analysis, five microorganisms were associated with risk of DLBCL. The genera *Terrisporobacter* (OR: 3.431, *p* = 0.049) andgenera *Oscillibacter* (OR: 2.406, *p* = 0.029) were associated with higher risk of DLBCL. Further, 27 plasma metabolites were identified as having a significant causal relationships with DLBCL, among which citrate levels had the most significant protective causal effect against DLBCL (*p* = 0.006), while glycosyl-N-tricosanoyl-sphingadienine levels was related to higher risk of DLBCL (*p* = 0.003). In addition, we identified 19 metabolite ratios with significant causal relationships to DLBCL, of which taurine/glutamate ratio had the most significant protective causal effect (*p* = 0.005), while the phosphoethanolamine/choline ratio was related to higher risk of DLBCL (*p* = 0.009). Reverse MR analysis did not reveal any significant causal influence of DLBCL on the above microbiota, metabolites, and metabolite ratios (*p* > 0.05). Sensitivity analyses revealed no significant heterogeneity or pleiotropy (*p* > 0.05).

**Conclusion:**

We present the first elucidation of the causal influence of microbiota and metabolites on DLBCL using MR methods, providing novel insights for potential targeting of specific microbiota or metabolites to prevent, assist in diagnosis, and treat DLBCL.

## 1 Introduction

Diffuse large B-cell lymphoma (DLBCL) is the most common subtype of invasive B-cell non-Hodgkin’s lymphoma (NHL), comprising approximately 40% of all malignant lymphomas (Alaggio et al., 2022). In terms of characteristics and clinical prognosis, DLBCL is a highly heterogeneous malignant tumor. In recent years, although patient treatment response rates have improved, more than 40% of patients with DLBCL continue to develop refractory disease with poor survival prognosis (Vodicka et al., 2022). Therefore, more study is needed to discover novel biomarkers for evaluating risk classification and guiding the optimization of personalized treatment for patients with DLBCL.

Notably, the number of genes derived from gut microbiota genomes is approximately 150 times greater than the number of genes in the human genome. Specific interactions occur between microorganisms and their metabolites and host cells (Yoo et al., 2020), which influence tumor occurrence and progression by inducing gene mutations, effecting the immune system, and altering metabolite levels, leading to inflammatory responses, and interfering with cell apoptosis and proliferation (Lu et al., 2022). Yuan et al. (2021) reported differential changes in gut microbiota between 25 patients with untreated DLBCL and healthy individuals using 16S rRNA gene sequencing. Further, Yoon et al. (2023) found that 189 patients with DLBCL exhibited microbiota dysbiosis, and that *Enterobacteriaceae* numbers were related to treatment efficacy and febrile neutropenia. Furthermore, Lin et al. (2023) detected correlations of the numbers of different microbes with disease characteristics and host immune cells in 35 patients with DLBCL. Previous studies have primarily relied on observing cross-sectional data or animal models; hence, although some associations between gut microbiota or metabolites and DLBCL have been proposed, it is difficult to effectively eliminate the influences of factors, such as age, region, habits, and lifestyle, limiting the determination of causal inference between various factors and DLBCL (Rinninella et al., 2019).

Metabolites are small molecule or compounds generated or transformed by enzymes during metabolic processes. The metabolism of cells driven to proliferate or die undergoes corresponding changes. There are reports that metabolic disorders in B-cell lymphoma may promote uncontrolled tumor cell proliferation, leading to the use of metabolic phenotypes as biomarkers for early cancer detection and/or treatment response (Vander and DeBerardinis, 2017). Alfaifi et al. (2023) summarized the diagnostic and prognostic significance of metabolic biomarkers in DLBCL using mass spectrometry and nuclear magnetic resonance techniques; however, few studies to date have reported the use of specific metabolic markers for DLBCL risk assessment.

Mendelian randomization (MR) integrates summary data from genome-wide association studies (GWAS) to determine causal influences of factors on outcomes, using genetic variation as instrumental variable, unaffected by confounding factors. MR analysis has been used to explore causal correlations between gut microbiota and various diseases, including autoimmune (Xu et al., 2021) and metabolic diseases (Sanna et al., 2019), as well as gastrointestinal tumors (Xie et al., 2023). In this study, we used MR analysis to investigate the potential causal effects of gut microbiota, plasma metabolites, and metabolite ratios on DLBCL, to provide data on potential early non-invasive diagnostic biomarkers and therapeutic targets for patients with DLBCL.

## 2 Methods

### 2.1 Dataset

The gut microbiota GWAS dataset was from the MiBioGen study, which explored genotype and 16S microbiome data from fecal samples from 18,340 participants (24 population cohorts) and conducted microbiota quantitative trait loci analysis to investigate the relationships between autosomal human genetic variation and the gut microbiome. And this study recorded 211 gut microbiota and 122,110 connected single nucleotide polymorphisms (SNPs) datasets, with a minimum classification level of genera. A total of 131 genera were determined with average abundance >1%, including 12 unknown genera (Kurilshikov et al., 2021). Thus, our study included 119 gut microbiota genera for analysis. The metamaterials and metamaterial rates GWAS dataset was from the Canadian Longitudinal Study on Aging (CLSA), which recorded 1091 metamaterials and 309 metamaterial rates from 8299 individuals (Raina et al., 2019). The DLBCL GWAS summary dataset was from the Integrative Epidemiology Unit Open GWAS project.^1^ The “finn-b-C3-DLBCL” dataset, which included 218,792 participants (209 cases and 218,583 controls) was selected.

### 2.2 Selection of instrumental variables

First, SNPs strongly correlated with gut microbiota, plasma metabolites, and metabolite ratios were identified as instrumental variables (IVs) (*p* < 1e-05). To guarantee stable correlations between IVs and exposure factors, weak IVs were filtered out, based on an *F* value [F = [R2/(R2–1)] [(N – K – 1)/K]] > 10. Second, to avoid the impact of linkage disequilibrium between genetic variations on the results and maintain the independence of selected IVs, thresholds of SNP linkage disequilibrium (r^2^) ≤ 0.001 and genetic spacing ≥10,000 kb were set. Third, to avoid IVs related to the results, those associated with DLBCL were removed (*p* < 0.05). In addition, palindromic SNPs were removed, to ensure that the influence of SNPs on exposure factors corresponded to the influence of a specific allele of SNP on outcomes.

### 2.3 MR analysis

Five MR methods [MR-Egger, inverse variance weighting (IVW), weighted median, simple mode, and weighted mode] were applied for analysis of the relationships of gut microbiota, plasma metabolites, and metabolite ratios with DLBCL. The IVW method uses meta-analysis integrated with Wald estimates for SNPs to evaluate the influence of exposure factors on an outcome. If there is no significant pleiotropy, the results of IVW will be unbiased (Burgess et al., 2016). MR Egger regression considers the potential heterogeneity of IVs and provides corrected estimates of causal effects, as well as an intercept term, to detect and correct bias (Bowden et al., 2015). The weighted median method provides a robust estimate of causal relationships, even when there are up to 50% invalid IVs (Hartwig et al., 2017). The weighted model method provides a comprehensive evaluation of the impact of different genotypes on outcomes by calculating the weighted average of each genotype, and better controls the influence of genotype frequency differences on the results, providing a robust and accurate analysis. If the results of analyses using these five MR methods were inconsistent, those obtained using the IVW method was used as the main evaluation result.

### 2.4 Sensitivity analysis

MR Egger and MR-Pleiotropy Residual Sum and Outlier (MR-PRESSO) tests were applied to examine pleiotropy and outliers, respectively; *p* > 0.05 indicated no significant pleiotropy. MR-PRESSO has higher accuracy than MR Egger analysis (Verbanck et al., 2018). Conchran’s Q-test was applied to assess the heterogeneity among IVs. The consistency of outliers and the overall results was analyzed using the leave-one-out method.

### 2.5 Reverse Mendelian randomization analysis

Reverse MR analysis was also conducted, using DLBCL as an exposure factor, and using gut microbiota, metabolites or metabolite ratios that were causally significantly related to DLBCL in MR analysis as outcomes, to explore whether DLBCL had a causal influence on microbiota and metabolites. Reverse MR analysis also used five methods (MR-Egger, IVW, weighted median, simple mode, and weighted mode), with pleiotropy and heterogeneity assessed using the MR Egger intercept test and the Cochran’s Q-test.

### 2.6 Statistical analysis

Statistical analyses were conducted in R software (version 4.1.2.). MR analysis was conducted using the R packages, TwoSampleMR (version 0.5.10) and MR-PRESSO (version 1.0). Visualize data using forest, scatter, funnel, and leave-one-out plots.

## 3 Results

### 3.1 Instrumental variables

We separately screened the IVs of 119 gut microbiota genera. According to the filtering criterion, *p* < 1e-05, IVs showing linkage disequilibrium in the microbiota (kb = 10,000 and *r*^2^ = 0.001) were removed. Further, IVs weakly correlated with exposure factors (*F* < 10) and possible confounding factors related to outcomes were also removed. Finally, 1,531 SNPs were included for analysis (Supplementary Excel S1). We also separately screened IVs for 1,091 plasma metabolite, and 309 metabolite ratios. According to the filtering criteria described above, 27,534 SNPs of plasma metabolite and 7,309 SNPs of metabolic ratios were included (Supplementary Excel S2).

### 3.2 MR analysis of gut microbiota

**Figure 1:**
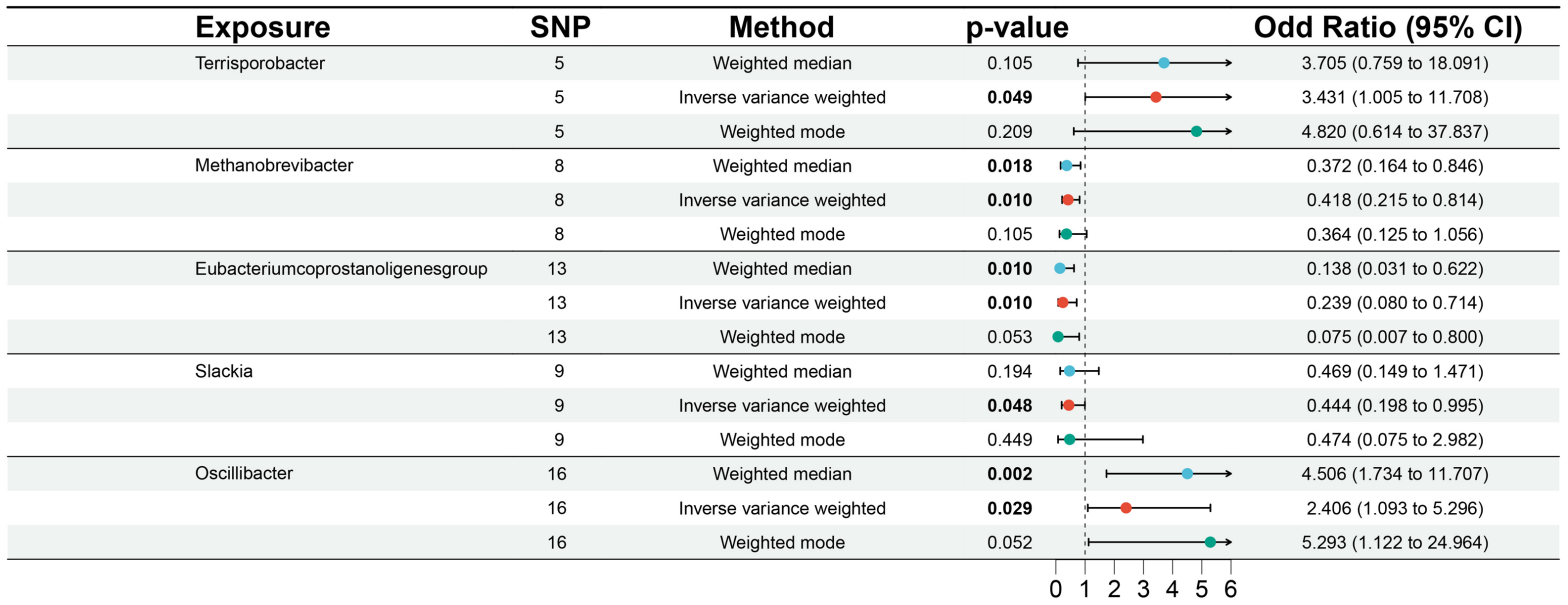
Associations of genetically predicted gut microbiota with diffuse large B-cell lymphoma (DLBCL) risk.

According to MR analysis using the IVW method, we detected causal relationships between 5 gut microbiota genera and DLBCL (Figure 1). Among them, the most significant was that the genus, *Oscillibacter*, was related to higher risk of DLBCL [odds ratio (OR): 2.406, 95 confidence interval (95%CI): 1.093–5.296, *p* = 0.029]. Further, application of the weighted median method yielded the same result (*p* = 0.002). Another gut microbiota genus, *Terrisporobacter*, was also related to higher risk of DLBCL (OR: 3.431, 95%CI: 1.005–11.708, *p* = 0.049). Conversely, the genera, *Methanobrevibacter*, *Eubacterium coprostanoligenes group*, and *Slackia* had causal protective effects against DLBCL (OR: 0.418, 95%CI: 0.215–0.814, *p* = 0.010; OR: 0.239, 95%CI: 0.080–0.714, *p* = 0.010; OR: 0.444, 95%CI: 0.198–0.995, *p* = 0.048). Meanwhile, according to the results of analysis using the weighted median method, the genera *Methanobrevibacter* and *Eubacterium coprostanoligenes group* were associated with low risk of DLBCL, similar to the results obtained using the IVW method (Table 1).

**Table 1:** Mendelian randomization analysis of associations between gut microbiota and diffuse large B-cell lymphoma.

Exposure	MR method	No. of SNP	*β*	SE	OR	95% CI		*p*-value
Terrisporobacter	Weighted mode	5	1.573	1.051	4.820	0.614	37.837	0.209
	Weighted median	5	1.310	0.809	3.705	0.759	18.091	0.106
	Inverse variance weighted	5	1.233	0.626	3.431	1.005	11.708	0.049
	MR Egger	5	1.086	2.168	2.961	0.042	207.593	0.652
	Simple mode	5	1.591	1.119	4.908	0.547	44.028	0.228
Methanobrevibacter	Weighted mode	8	−1.011	0.544	0.364	0.125	1.056	0.105
	Weighted median	8	−0.988	0.419	0.372	0.164	0.846	0.018
	Inverse variance weighted	8	−0.872	0.340	0.418	0.215	0.814	0.010
	MR Egger	8	−0.010	1.346	0.990	0.071	13.852	0.995
	Simple mode	8	−1.002	0.582	0.367	0.117	1.147	0.128
*Eubacterium coprostanoligenes* group	Weighted mode	13	−2.589	1.207	0.075	0.007	0.800	0.053
	Weighted median	13	−1.978	0.766	0.138	0.031	0.622	0.010
	Inverse variance weighted	13	−1.431	0.559	0.239	0.080	0.714	0.010
	MR Egger	13	−3.370	2.191	0.034	<0.001	2.521	0.152
	Simple mode	13	−2.611	1.312	0.073	0.006	0.961	0.070
Slackia	Weighted mode	9	−0.747	0.939	0.474	0.075	2.982	0.449
	Weighted median	9	−0.757	0.583	0.469	0.149	1.471	0.194
	Inverse variance weighted	9	−0.811	0.411	0.444	0.198	0.995	0.048
	MR Egger	9	−3.210	1.912	0.040	0.001	1.711	0.137
	Simple mode	9	−0.736	0.849	0.479	0.091	2.527	0.411
Oscillibacter	Weighted mode	16	1.666	0.791	5.293	1.122	24.964	0.052
	Weighted median	16	1.505	0.487	4.506	1.734	11.707	0.002
	Inverse variance weighted	16	0.878	0.402	2.406	1.093	5.296	0.029
	MR Egger	16	0.797	1.450	2.219	0.130	38.002	0.591
	Simple mode	16	1.666	0.786	5.293	1.134	24.709	0.051

MR, Mendelian randomization; SNP, single nucleotide polymorphisms; β, Beta; SE, standard error; OR, odds ratio; CI, confidence interval.

**Figure 2:**
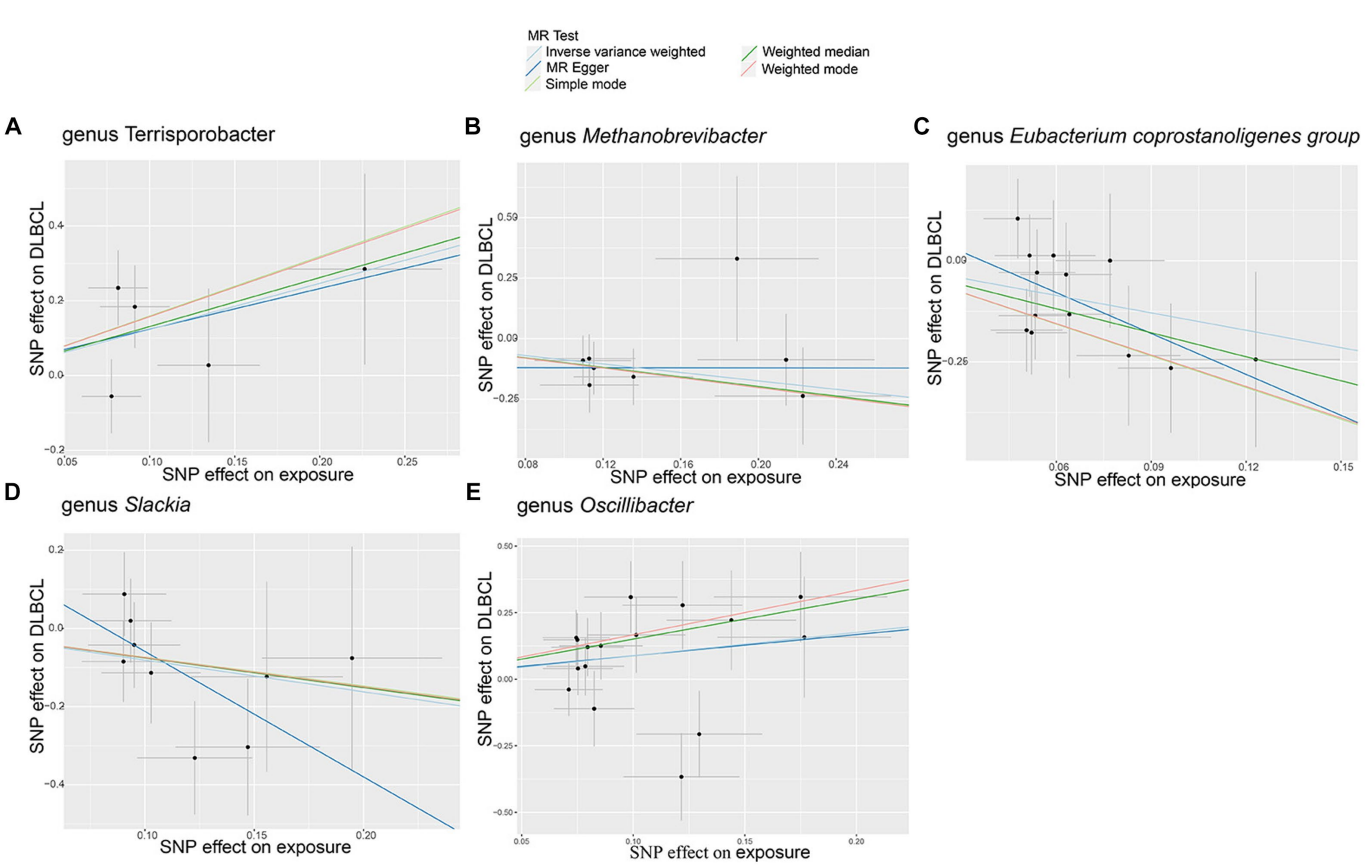
Scatter plots of causal estimates for genetically predicted gut microbiota on diffuse large B-cell lymphoma (DLBCL) risk. **(A)** genus *Terrisporobacter*; **(B)** genus *Methanobrevibacter*; **(C)** genus *Eubacterium coprostanoligenes group*; **(D)** genus *Slackia*; **(E)** genus *Oscillibacter*.

In this study, no significant pleiotropy or outliers were detected using the MR Egger intercept test, MR-PRESSO test (Supplementary Table S1, *p* > 0.05), or scatter plot (Figure 2). Further, no significant heterogeneity was detected among the selected SNPs using the Cochran’s Q-test (Supplementary Table S1, *p* > 0.05) or funnel plot analysis (Supplementary Figure S1). In addition, the stability of MR results was analyzed by leave-one-out analysis (Supplementary Figure S2).

### 3.3 MR analysis of plasma metabolites

**Figure 3:**
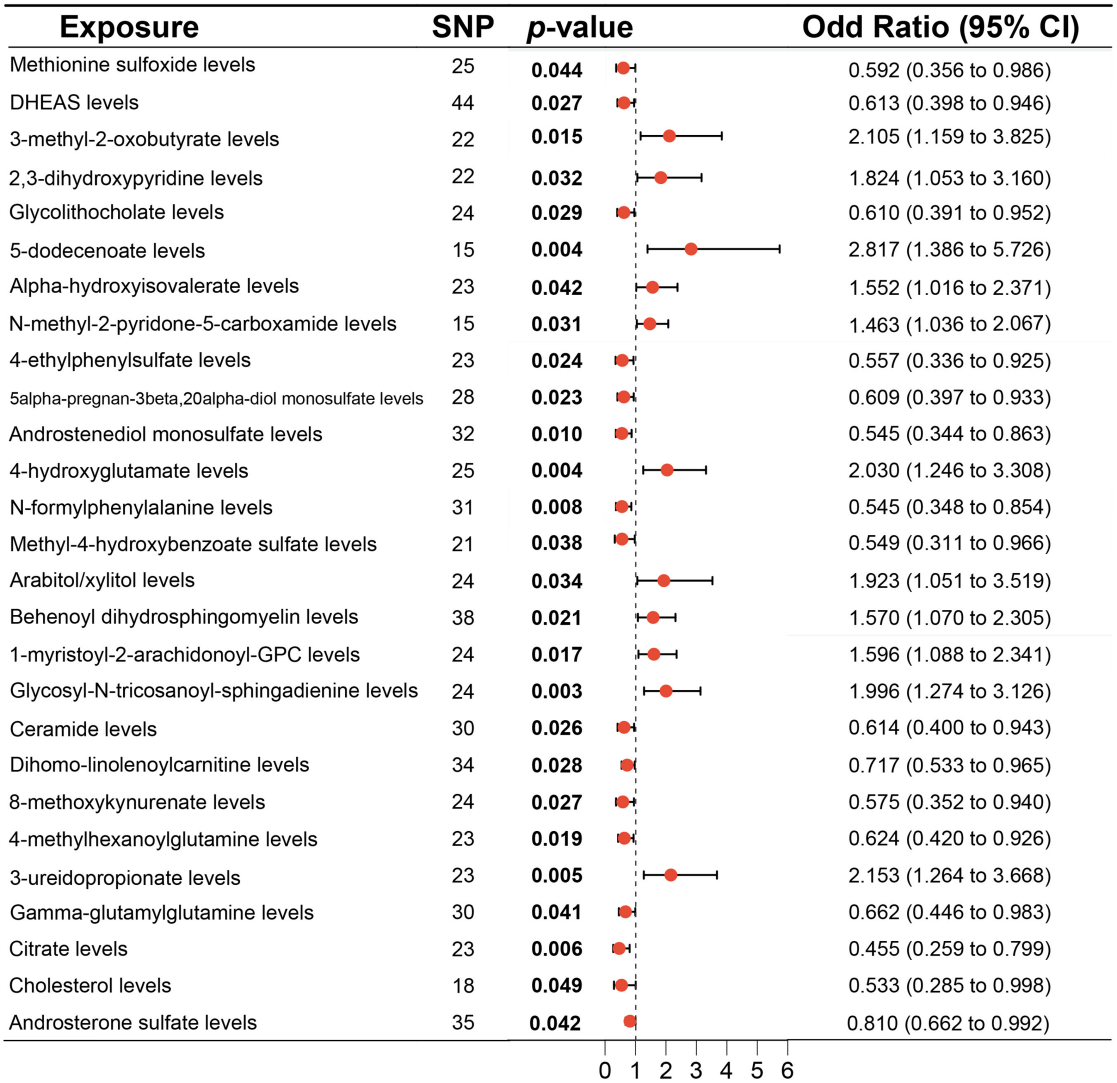
Associations of genetically predicted plasma metabolites with diffuse large B-cell lymphoma (DLBCL) risk analyzed using the inverse variance weighting (IVW) method.

According to MR analysis by the IVW method, associations between 27 plasma metabolites and the risk of DLBCL were identified (Figure 3). The top five most significant metabolites associated with high risk of DLBCL were levels of glycosyl-N-tricosanoyl-sphingadienine (*p* = 0.003), 5-dodecenoate (*p* = 0.004), 4-hydroxyglutamate (*p* = 0.004), 3-ureidopropionate (*p* = 0.005), and 3-methyl-2-oxobutyrate (*p* = 0.015). Further, the top three metabolites were significantly correlated with causal protective effects against DLBCL, including those of citrate (*p* = 0.006), N-formylphenylalanine (*p* = 0.008), and androstenediol monosulfate (*p* = 0.010). Analysis using the weighted median method indicated that DHEAS, glycolithocolate, androstenediol monosulfate, 4-hydroxyglutamate, and methyl-4-hydroxybenzoate sulfate were associated with DLBCL, similar to the results produced using the IVW method (Table 2).

**Table 2:** Mendelian randomization analysis of associations between plasma metabolites and diffuse large B-cell lymphoma.

Exposure	MR method	No. of SNP	*β*	SE	OR	95% CI		*p*-value
Methionine sulfoxide levels	MR Egger	25	−0.966	0.562	0.381	0.127	1.145	0.099
	Weighted median	25	−0.533	0.347	0.587	0.297	1.159	0.125
	Inverse variance weighted	25	−0.524	0.260	0.592	0.356	0.986	0.044
	Weighted mode	25	−0.981	0.629	0.375	0.109	1.286	0.132
	Simple mode	25	−0.146	0.658	0.864	0.238	3.138	0.827
DHEAS levels	MR Egger	44	−0.396	0.399	0.673	0.308	1.472	0.327
	Weighted median	44	−0.713	0.347	0.490	0.248	0.969	0.040
	Inverse variance weighted	44	−0.489	0.221	0.613	0.398	0.946	0.027
	Weighted mode	44	−0.781	0.338	0.459	0.2368	0.889	0.026
	Simple mode	44	−0.959	0.552	0.383	0.130	1.130	0.089
3-methyl-2-oxobutyrate levels	MR Egger	22	0.001	0.718	1.001	0.245	4.085	0.999
	Weighted median	22	0.581	0.417	1.789	0.790	4.051	0.163
	Inverse variance weighted	22	0.744	0.305	2.105	1.159	3.825	0.015
	Weighted mode	22	0.284	0.740	1.329	0.311	5.671	0.705
	Simple mode	22	0.203	0.800	1.226	0.256	5.878	0.802
2,3-dihydroxypyridine levels	MR Egger	22	1.12	0.983	3.072	0.447	21.102	0.267
	Weighted median	22	0.536	0.388	1.709	0.799	3.655	0.167
	Inverse variance weighted	22	0.601	0.280	1.82	1.053	3.160	0.032
	Weighted mode	22	0.502	0.604	1.652	0.506	5.397	0.415
	Simple mode	22	0.519	0.666	1.680	0.455	6.201	0.445
Glycolithocholate levels	MR Egger	24	−0.597	0.422	0.552	0.242	1.260	0.172
	Weighted median	24	−0.690	0.322	0.502	0.267	0.942	0.032
	Inverse variance weighted	24	−0.494	0.227	0.610	0.391	0.952	0.029
	Weighted mode	24	−0.855	0.449	0.425	0.176	1.024	0.069
	Simple mode	24	−0.870	0.578	0.419	0.135	1.3011	0.146
5-dodecenoate levels	MR Egger	15	0.655	0.786	1.924	0.413	8.974	0.420
	Weighted median	15	0.467	0.523	1.594	0.572	4.442	0.372
	Inverse variance weighted	15	1.036	0.362	2.817	1.386	5.726	0.004
	Weighted mode	15	0.478	0.607	1.612	0.491	5.294	0.444
	Simple mode	15	1.981	0.888	7.247	1.271	41.332	0.043
Alpha-hydroxyisovalerate levels	MR Egger	23	0.519	0.435	1.680	0.716	3.940	0.247
	Weighted median	23	0.195	0.307	1.215	0.666	2.217	0.526
	Inverse variance weighted	23	0.439	0.216	1.552	1.016	2.371	0.042
	Weighted mode	23	0.244	0.328	1.276	0.671	2.427	0.465
	Simple mode	23	0.831	0.562	2.295	0.763	6.905	0.153
N-methyl-2-pyridone-5-carboxamide levels	MR Egger	15	0.352	0.233	1.422	0.901	2.243	0.154
	Weighted median	15	0.384	0.566	1.468	0.484	4.450	0.497
	Inverse variance weighted	15	0.381	0.176	1.463	1.036	2.067	0.031
	Weighted mode	15	0.404	0.229	1.497	0.956	2.344	0.099
	Simple mode	15	0.695	0.586	2.003	0.636	6.313	0.255
4-ethylphenylsulfate levels	MR Egger	23	−0.531	0.458	0.588	0.240	1.444	0.260
	Weighted median	23	−0.383	0.385	0.682	0.320	1.451	0.320
	Inverse variance weighted	23	−0.585	0.258	0.557	0.336	0.925	0.024
	Weighted mode	23	−0.262	0.467	0.769	0.308	1.920	0.580
	Simple mode	23	−0.308	0.643	0.735	0.208	2.591	0.636
5alpha-pregnan-3beta,20alpha-diol monosulfate levels	MR Egger	28	−0.608	0.474	0.544	0.215	1.378	0.211
	Weighted median	28	−0.297	0.324	0.743	0.394	1.402	0.360
	Inverse variance weighted	28	−0.496	0.218	0.609	0.397	0.933	0.023
	Weighted mode	28	−0.398	0.406	0.672	0.303	1.489	0.336
	Simple mode	28	−1.052	0.590	0.349	0.110	1.110	0.086
Androstenediol monosulfate levels	MR Egger	32	−0.579	0.413	0.560	0.249	1.259	0.171
	Weighted median	32	−0.773	0.355	0.461	0.230	0.926	0.030
	Inverse variance weighted	32	−0.607	0.235	0.545	0.344	0.863	0.010
	Weighted mode	32	−0.766	0.372	0.465	0.224	0.963	0.048
	Simple mode	32	−0.815	0.603	0.443	0.136	1.442	0.186
4-hydroxyglutamate levels	MR Egger	25	0.028	0.521	1.028	0.371	2.852	0.958
	Weighted median	25	0.874	0.346	2.396	1.217	4.717	0.011
	Inverse variance weighted	25	0.708	0.249	2.030	1.2461	3.308	0.004
	Weighted mode	25	0.925	0.505	2.522	0.936	6.791	0.080
	Simple mode	25	0.662	0.603	1.939	0.595	6.318	0.283
N-formylphenylalanine levels	MR Egger	31	−1.148	0.545	0.317	0.109	0.922	0.044
	Weighted median	31	−0.313	0.341	0.731	0.374	1.428	0.359
	Inverse variance weighted	31	−0.606	0.229	0.545	0.348	0.854	0.008
	Weighted mode	31	−0.175	0.619	0.839	0.249	2.826	0.779
	Simple mode	31	−0.175	0.646	0.839	0.237	2.977	0.788
Methyl-4-hydroxybenzoate sulfate levels	MR Egger	21	−1.311	0.580	0.269	0.087	0.839	0.036
	Weighted median	21	−0.823	0.379	0.439	0.209	0.923	0.030
	Inverse variance weighted	21	−0.601	0.289	0.549	0.311	0.966	0.038
	Weighted mode	21	−0.833	0.535	0.435	0.152	1.241	0.135
	Simple mode	21	−1.059	0.640	0.347	0.099	1.216	0.114
Arabitol/xylitol levels	MR Egger	24	0.453	0.764	1.573	0.352	7.034	0.559
	Weighted median	24	0.575	0.421	1.777	0.778	4.059	0.172
	Inverse variance weighted	24	0.654	0.308	1.923	1.051	3.519	0.034
	Weighted mode	24	0.272	0.710	1.312	0.326	5.281	0.706
	Simple mode	24	0.050	0.804	1.051	0.217	5.085	0.951
Behenoyl dihydrosphingomyelin levels	MR Egger	38	0.427	0.447	1.533	0.638	3.680	0.346
	Weighted median	38	0.497	0.302	1.643	0.909	2.969	0.100
	Inverse variance weighted	38	0.451	0.196	1.570	1.070	2.305	0.021
	Weighted mode	38	0.700	0.498	2.013	0.758	5.346	0.169
	Simple mode	38	0.406	0.569	1.500	0.492	4.575	0.480
1-myristoyl-2-arachidonoyl-GPC levels	MR Egger	24	0.917	0.334	2.502	1.299	4.818	0.012
	Weighted median	24	0.409	0.263	1.505	0.898	2.521	0.121
	Inverse variance weighted	24	0.467	0.195	1.596	1.088	2.341	0.017
	Weighted mode	24	0.460	0.272	1.584	0.929	2.700	0.105
	Simple mode	24	0.4210	0.610	1.524	0.461	5.036	0.497
Glycosyl-N-tricosanoyl-sphingadienine levels	MR Egger	24	0.389	0.514	1.475	0.538	4.044	0.458
	Weighted median	24	0.659	0.339	1.933	0.995	3.756	0.052
	Inverse variance weighted	24	0.691	0.229	1.996	1.274	3.126	0.003
	Weighted mode	24	0.742	0.461	2.100	0.851	5.185	0.121
	Simple mode	24	0.755	0.610	2.128	0.644	7.030	0.228
Ceramide levels	MR Egger	30	−0.765	0.506	0.466	0.173	1.255	0.142
	Weighted median	30	−0.315	0.318	0.730	0.391	1.361	0.322
	Inverse variance weighted	30	−0.487	0.219	0.614	0.400	0.943	0.026
	Weighted mode	30	−0.339	0.4449	0.713	0.2988	1.702	0.452
	Simple mode	30	−0.446	0.588	0.640	0.202	2.025	0.454
Dihomo-linolenoylcarnitine levels	MR Egger	34	−0.128	0.269	0.879	0.520	1.489	0.636
	Weighted median	34	−0.336	0.207	0.715	0.477	1.072	0.105
	Inverse variance weighted	34	−0.333	0.151	0.717	0.533	0.965	0.028
	Weighted mode	34	−0.295	0.226	0.745	0.478	1.161	0.202
	Simple mode	34	−0.522	0.395	0.593	0.274	1.286	0.195
8-methoxykynurenate levels	MR Egger	24	−0.138	0.655	0.871	0.241	3.146	0.835
	Weighted median	24	−0.463	0.327	0.630	0.332	1.195	0.157
	Inverse variance weighted	24	−0.553	0.251	0.575	0.352	0.940	0.027
	Weighted mode	24	−0.455	0.511	0.634	0.233	1.728	0.383
	Simple mode	24	−0.500	0.573	0.606	0.197	1.863	0.391
4-methylhexanoylglutamine levels	MR Egger	23	−0.170	0.388	0.844	0.394	1.805	0.666
	Weighted median	23	−0.391	0.293	0.676	0.381	1.201	0.182
	Inverse variance weighted	23	−0.472	0.202	0.624	0.420	0.926	0.019
	Weighted mode	23	−0.442	0.370	0.642	0.311	1.327	0.245
	Simple mode	23	−0.645	0.523	0.525	0.188	1.463	0.231
3-ureidopropionate levels	MR Egger	23	0.357	0.535	1.429	0.501	4.077	0.511
	Weighted median	23	0.604	0.380	1.830	0.869	3.857	0.112
	Inverse variance weighted	23	0.767	0.272	2.153	1.264	3.668	0.005
	Weighted mode	23	0.435	0.493	1.544	0.588	4.059	0.388
	Simple mode	23	1.082	0.629	2.950	0.8596	10.124	0.100
Gamma-glutamylglutamine levels	MR Egger	30	−0.489	0.407	0.613	0.276	1.360	0.239
	Weighted median	30	−0.361	0.283	0.697	0.400	1.215	0.203
	Inverse variance weighted	30	−0.412	0.202	0.662	0.446	0.983	0.041
	Weighted mode	30	−0.239	0.351	0.787	0.395	1.567	0.501
	Simple mode	30	−0.078	0.464	0.925	0.372	2.299	0.868
Citrate levels	MR Egger	23	−0.818	0.702	0.442	0.112	1.747	0.257
	Weighted median	23	−0.697	0.384	0.498	0.235	1.058	0.070
	Inverse variance weighted	23	−0.788	0.288	0.455	0.259	0.799	0.006
	Weighted mode	23	−1.090	0.735	0.336	0.080	1.419	0.152
	Simple mode	23	−1.071	0.751	0.343	0.079	1.495	0.168
Cholesterol levels	MR Egger	18	−0.750	0.643	0.472	0.134	1.666	0.261
	Weighted median	18	−0.466	0.451	0.628	0.259	1.519	0.302
	Inverse variance weighted	18	−0.629	0.320	0.533	0.285	0.998	0.049
	Weighted mode	18	−0.453	0.625	0.636	0.187	2.165	0.479
	Simple mode	18	−0.534	0.708	0.586	0.146	2.349	0.461
Androsterone sulfate levels	MR Egger	35	−0.206	0.125	0.813	0.637	1.039	0.108
	Weighted median	35	−0.194	0.112	0.824	0.661	1.026	0.084
	Inverse variance weighted	35	−0.210	0.103	0.810	0.662	0.992	0.042
	Weighted mode	35	−0.185	0.107	0.831	0.674	1.026	0.094
	Simple mode	35	−0.336	0.398	0.714	0.327	1.560	0.405

MR, Mendelian randomization; SNP, single nucleotide polymorphisms; β, Beta; SE, standard error; OR, odds ratio; CI, confidence interval.

**Figure 4:**
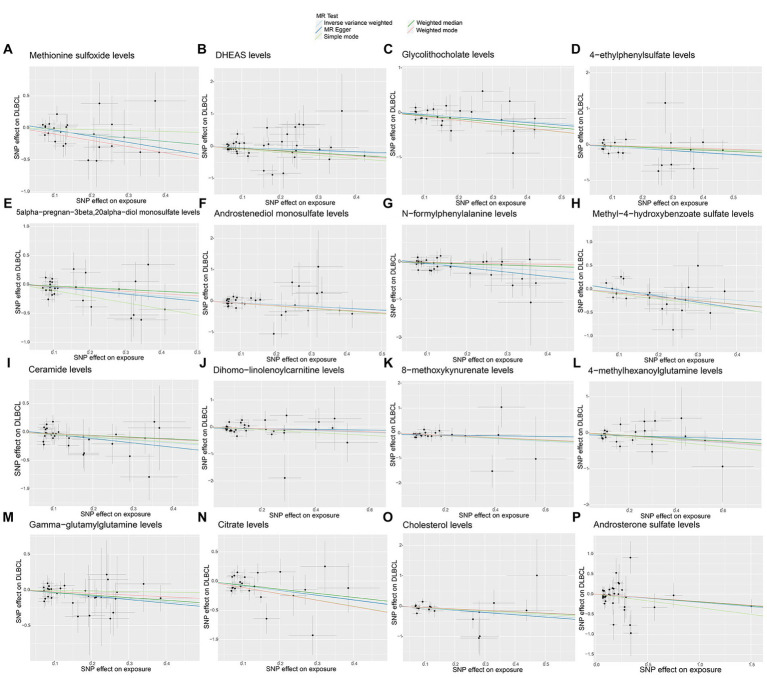
Scatter plots of causal estimates for genetically predicted plasma metabolites protective against diffuse large B-cell lymphoma (DLBCL) risk. **(A)** Methionine sulfoxide levels; **(B)** DHEAS levels; **(C)** Glycolithocholate levels; **(D)** 4-ethylphenylsulfate levels; **(E)** 5alpha-pregnan-3beta,20alpha-diol monosulfate levels; **(F)** Androstenediol monosulfate levels; **(G)** N-formylphenylalanine levels; **(H)** Methyl-4-hydroxybenzoate sulfate levels; **(I)** Ceramide levels; **(J)** Dihomo-linolenoylcarnitine levels; **(K)** 8-methoxykynurenate levels; **(L)** 4-methylhexanoylglutamine levels; **(M)** Gamma-glutamylglutamine levels; **(N)** Citrate levels; **(O)** Cholesterol levels; **(P)** Androsterone sulfate levels.

**Figure 5:**
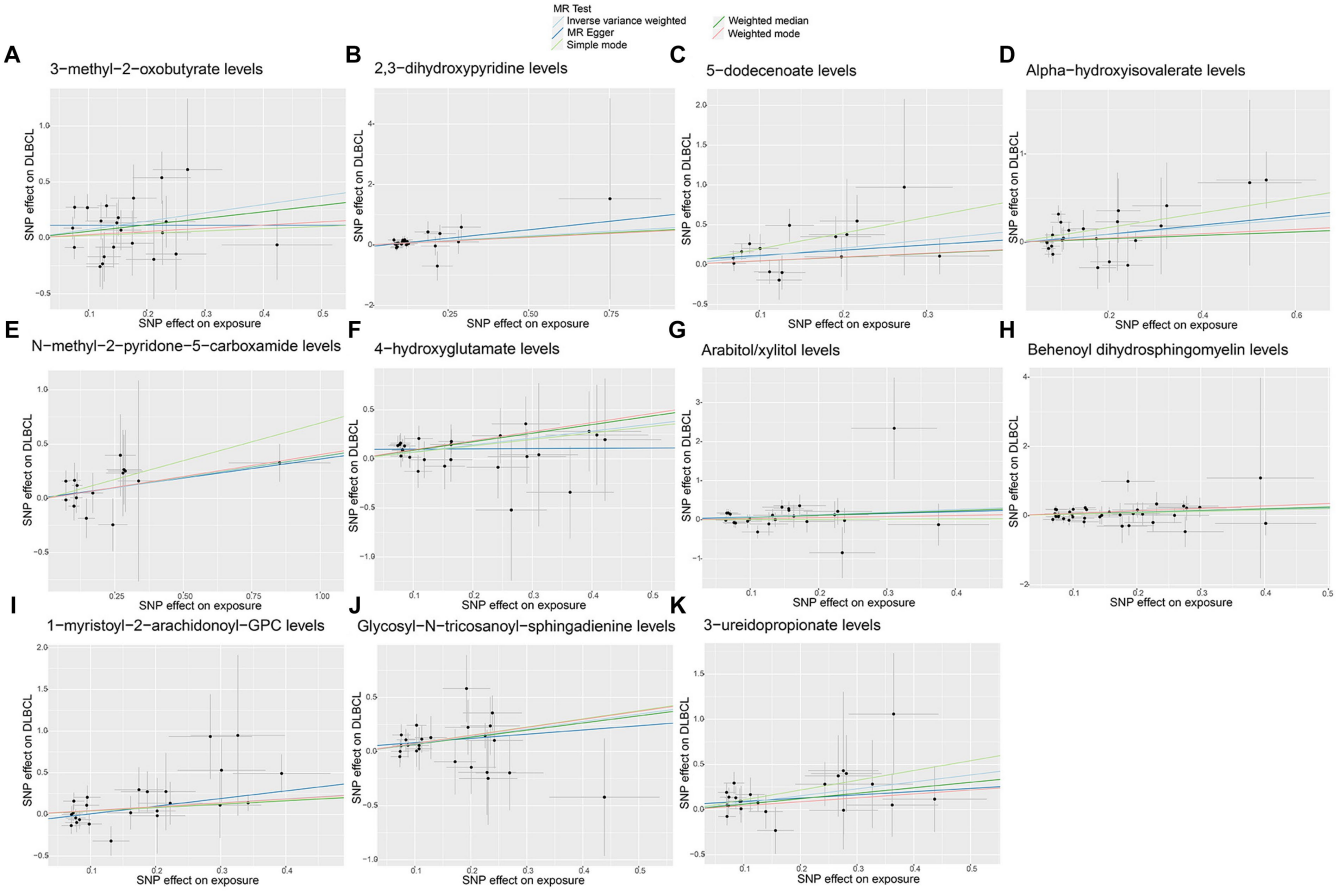
Scatter plots of causal estimates for genetically predicted plasma metabolites contributing to diffuse large B-cell lymphoma (DLBCL) risk. **(A)** 3-methyl-2-oxobutyrate levels; **(B)** 2,3-dihydroxypyridine levels; **(C)** 5-dodecenoate levels; **(D)** Alpha-hydroxyisovalerate levels; **(E)** N-methyl-2-pyridone-5-carboxamide levels; **(F)** 4-hydroxyglutamate levels; **(G)** Arabitol/xylitol levels; **(H)** Behenoyl dihydrosphingomyelin levels; **(I)** 1-myristoyl-2-arachidonoyl-GPC levels; **(J)** Glycosyl-N-tricosanoyl-sphingadienine levels; **(K)** 3-ureidopropionate levels.

No significant pleiotropy or outliers were detected using the MR Egger intercept test, MR-PRESSO test (Supplementary Table S2, *p* > 0.05), or scatter plot (Figures 4, 5). Further, there were no significant heterogeneity (*p* > 0.05) among selected SNPs, according to the Cochran’s Q-test (Supplementary Table S2, *p* > 0.05) and funnel plots analysis (Supplementary Figures S3, S4). The stability of MR results was analyzed using leave-one-out analysis (Supplementary Figure S5).

### 3.4 MR analysis of metabolite ratio

**Figure 6:**
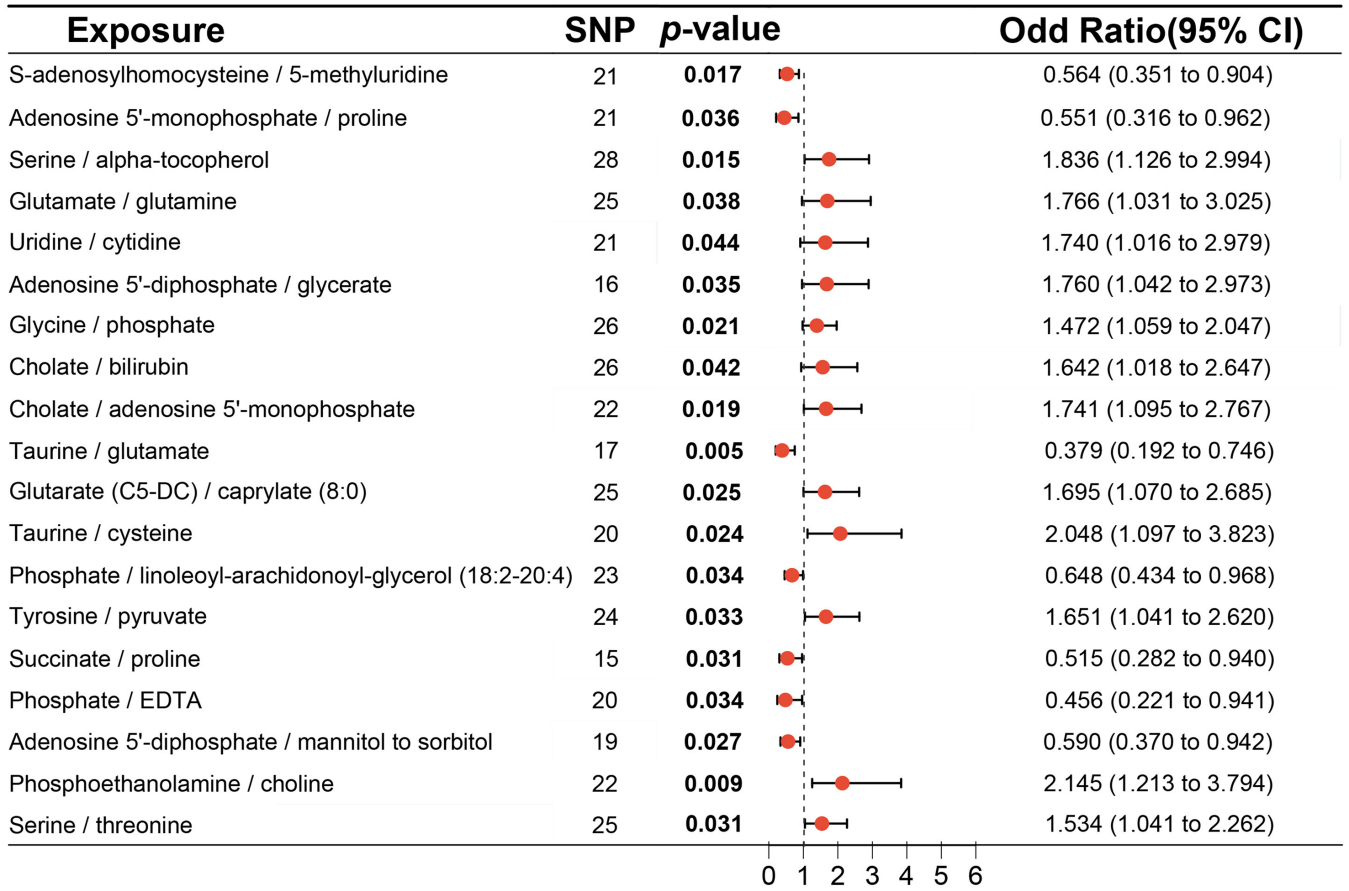
Associations of genetically predicted metabolite ratios with diffuse large B-cell lymphoma (DLBCL) risk analyzed using inverse the variance weighting (IVW) method.

MR analysis using the IVW method indentified 19 metabolite ratios as associated with the risk of DLBCL (Figure 6). Among them, serine/alpha tocopherol, glutamate/glutamine, uridine/cytidine, adenosine 5′-diphosphate/glycerate, glycine/phosphate, cholate/bilirubin, cholate/adenosine 5′-monophosphate, glutarate (C5-DC)/caprylate (8:0), taurine/cysteine, tyrosine/pyruvate, phosphoethanolamine/choline, and serine/threonine were associated with a higher risk of DLBCL (*p* < 0.05). Notably, s-adenosylhomocysteine/5-methyluridine, adenosine 5′-monophosphate/proline, taurine/glutamate, phosphate/linoleoyl-arachidonoyl-glycerol (18:2–20:4), succinate/proline, phosphate/EDTA, and adenosine 5′–diphosphate/mannitol to sorbitol had a causal protective effects against DLBCL (*p* < 0.05). Further, analysis using the weighted median method indicated that s-adenosylhomocysteine/5-methyluridine, taurine/glutamate, and phosphate/EDTA were associated with low risk of DLBCL, consistent with the results generated by IVW analysis (Table 3).

**Table 3:** Mendelian randomization analysis of association between metabolite ratios and diffuse large B-cell lymphoma.

Exposure	MR method	No. of SNP	*β*	SE	OR	95% CI		*p*-value
S-adenosylhomocysteine/ 5-methyluridine	MR Egger	21	−0.300	0.551	0.741	0.252	2.180	0.592
	Weighted median	21	−0.882	0.316	0.414	0.223	0.769	0.005
	Inverse variance weighted	21	−0.573	0.241	0.564	0.351	0.904	0.017
	Weighted mode	21	−0.767	0.329	0.464	0.244	0.885	0.030
	Simple mode	21	−0.767	0.493	0.464	0.177	1.221	0.136
Adenosine 5′-monophosphate / proline	MR Egger	21	−0.923	0.612	0.397	0.120	1.320	0.148
	Weighted median	21	−0.565	0.409	0.568	0.255	1.268	0.168
	Inverse variance weighted	21	−0.596	0.284	0.551	0.316	0.962	0.036
	Weighted mode	21	−1.104	0.736	0.331	0.078	1.403	0.149
	Simple mode	21	−0.768	0.792	0.464	0.098	2.190	0.344
Serine / alpha-tocopherol	MR Egger	28	1.414	0.546	4.111	1.409	11.995	0.016
	Weighted median	28	0.597	0.328	1.817	0.956	3.453	0.068
	Inverse variance weighted	28	0.608	0.249	1.836	1.126	2.994	0.015
	Weighted mode	28	0.650	0.495	1.916	0.726	5.055	0.200
	Simple mode	28	0.296	0.616	1.344	0.402	4.492	0.635
Glutamate / glutamine	MR Egger	25	1.293	0.589	3.642	1.148	11.559	0.039
	Weighted median	25	0.685	0.364	1.984	0.973	4.048	0.059
	Inverse variance weighted	25	0.569	0.274	1.766	1.031	3.025	0.038
	Weighted mode	25	0.810	0.596	2.248	0.698	7.233	0.187
	Simple mode	25	0.693	0.691	1.999	0.516	7.749	0.326
Uridine / cytidine	MR Egger	21	0.625	0.582	1.868	0.597	5.845	0.296
	Weighted median	21	0.109	0.391	1.115	0.518	2.400	0.781
	Inverse variance weighted	21	0.554	0.274	1.740	1.016	2.980	0.044
	Weighted mode	21	0.075	0.572	1.078	0.351	3.309	0.897
	Simple mode	21	0.177	0.623	1.194	0.352	4.050	0.779
Adenosine 5′-diphosphate/ glycerate	MR Egger	16	0.827	0.769	2.286	0.506	10.326	0.301
	Weighted median	16	0.644	0.376	1.904	0.911	3.979	0.087
	Inverse variance weighted	16	0.565	0.268	1.760	1.042	2.973	0.035
	Weighted mode	16	1.016	0.647	2.762	0.777	9.819	0.137
	Simple mode	16	1.003	0.713	2.725	0.674	11.023	0.180
Glycine / phosphate	MR Egger	26	0.350	0.253	1.418	0.864	2.329	0.180
	Weighted median	26	0.154	0.197	1.167	0.793	1.717	0.434
	Inverse variance weighted	26	0.387	0.168	1.472	1.059	2.047	0.021
	Weighted mode	26	0.205	0.197	1.227	0.833	1.807	0.310
	Simple mode	26	0.512	0.5613	1.669	0.556	5.015	0.370
Cholate / bilirubin	MR Egger	26	0.168	0.497	1.183	0.447	3.133	0.738
	Weighted median	26	0.481	0.336	1.617	0.837	3.124	0.152
	Inverse variance weighted	26	0.496	0.244	1.641	1.018	2.647	0.042
	Weighted mode	26	0.501	0.344	1.651	0.841	3.241	0.158
	Simple mode	26	0.785	0.549	2.193	0.748	6.435	0.165
Cholate / adenosine 5′-monophosphate	MR Egger	22	0.252	0.430	1.286	0.554	2.989	0.565
	Weighted median	22	0.508	0.346	1.661	0.843	3.273	0.142
	Inverse variance weighted	22	0.554	0.236	1.741	1.095	2.767	0.019
	Weighted mode	22	0.450	0.477	1.569	0.616	3.994	0.356
	Simple mode	22	1.392	0.570	4.023	1.315	12.307	0.024
Taurine / glutamate	MR Egger	17	−1.371	0.862	0.254	0.047	1.375	0.132
	Weighted median	17	−0.973	0.472	0.378	0.150	0.953	0.039
	Inverse variance weighted	17	−0.971	0.346	0.379	0.192	0.746	0.005
	Weighted mode	17	−1.299	0.748	0.273	0.063	1.181	0.102
	Simple mode	17	−1.375	0.750	0.253	0.058	1.098	0.085
Glutarate (C5-DC) / caprylate (8:0)	MR Egger	25	0.868	0.418	2.383	1.050	5.406	0.049
	Weighted median	25	0.626	0.370	1.871	0.906	3.861	0.090
	Inverse variance weighted	25	0.528	0.235	1.695	1.070	2.685	0.025
	Weighted mode	25	0.623	0.379	1.864	0.888	3.916	0.113
		25	0.491	0.544	1.634	0.5636	4.744	0.375
Taurine / cysteine	MR Egger	20	0.038	0.701	1.039	0.263	4.105	0.957
	Weighted median	20	0.763	0.446	2.144	0.894	5.142	0.087
	Inverse variance weighted	20	0.717	0.318	2.048	1.097	3.823	0.024
	Weighted mode	20	0.881	0.677	2.412	0.639	9.099	0.209
	Simple mode	20	0.881	0.743	2.412	0.563	10.341	0.250
Phosphate / linoleoyl-arachidonoyl-glycerol (18:2–20:4)	MR Egger	23	−0.621	0.412	0.537	0.240	1.204	0.146
	Weighted median	23	−0.528	0.290	0.590	0.335	1.041	0.068
	Inverse variance weighted	23	−0.433	0.204	0.648	0.434	0.968	0.034
	Weighted mode	23	−0.512	0.306	0.599	0.329	1.093	0.109
	Simple mode	23	−0.482	0.539	0.617	0.215	1.775	0.380
Tyrosine / pyruvate	MR Egger	24	0.529	0.426	1.697	0.736	3.914	0.228
	Weighted median	24	0.442	0.364	1.556	0.763	3.175	0.224
	Inverse variance weighted	24	0.501	0.236	1.651	1.041	2.620	0.033
	Weighted mode	24	0.456	0.376	1.578	0.756	3.29	0.237
	Simple mode	24	0.245	0.551	1.277	0.434	3.763	0.661
Succinate / proline	MR Egger	15	−0.752	0.6205	0.471	0.140	1.587	0.246
	Weighted median	15	−0.493	0.432	0.611	0.262	1.425	0.254
	Inverse variance weighted	15	−0.664	0.307	0.515	0.282	0.940	0.031
	Weighted mode	15	−0.322	0.602	0.724	0.223	2.356	0.601
	Simple mode	15	−0.091	0.718	0.912	0.223	3.731	0.900
Phosphate / EDTA	MR Egger	20	−0.503	1.217	0.605	0.056	6.571	0.684
	Weighted median	20	−1.265	0.487	0.282	0.109	0.732	0.009
	Inverse variance weighted	20	−0.785	0.370	0.456	0.221	0.941	0.034
	Weighted mode	20	−1.570	0.603	0.208	0.064	0.678	0.018
	Simple mode	20	−1.570	0.730	0.208	0.050	0.870	0.045
Adenosine 5′-diphosphate / mannitol to sorbitol	MR Egger	19	−0.454	0.574	0.635	0.206	1.957	0.440
	Weighted median	19	−0.497	0.337	0.608	0.314	1.178	0.140
	Inverse variance weighted	19	−0.527	0.238	0.590	0.370	0.942	0.027
	Weighted mode	19	−0.972	0.598	0.379	0.117	1.221	0.121
	Simple mode	19	−0.972	0.647	0.379	0.107	1.344	0.150
Phosphoethanolamine / choline	MR Egger	22	1.131	0.829	3.098	0.610	15.727	0.188
	Weighted median	22	0.593	0.412	1.809	0.808	4.053	0.150
	Inverse variance weighted	22	0.763	0.291	2.145	1.213	3.794	0.009
	Weighted mode	22	0.235	0.638	1.265	0.362	4.416	0.716
	Simple mode	22	0.983	0.714	2.673	0.660	10.825	0.183
Serine / threonine	MR Egger	25	0.415	0.405	1.514	0.685	3.347	0.316
	Weighted median	25	0.311	0.291	1.365	0.772	2.412	0.284
	Inverse variance weighted	25	0.428	0.198	1.534	1.041	2.262	0.031
	Weighted mode	25	0.385	0.276	1.470	0.856	2.525	0.175
	Simple mode	25	0.566	0.472	1.762	0.698	4.448	0.242

MR, Mendelian randomization; SNP, single nucleotide polymorphisms; β, Beta; SE, standard error; OR, odds ratio; CI, confidence interval.

**Figure 7:**
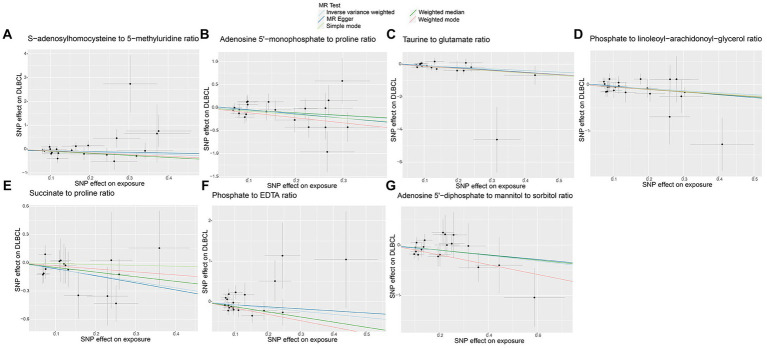
Scatter plots of causal estimates for genetically predicted metabolite ratios protective against diffuse large B-cell lymphoma (DLBCL) risk. **(A)** S-adenosylhomocysteine to 5-methyluridine ratio; **(B)** Adenosine 5’-monophosphate to proline ratio; **(C)** Taurine to glutamate ratio; **(D)** Phosphate to linoleoyl-arachidonoyl-glycerol ratio; **(E)** Succinate to proline ratio; **(F)** Phosphate to EDTA ratio; **(G)** Adenosine 5’-diphosphate to mannitol to sorbitol ratio.

**Figure 8:**
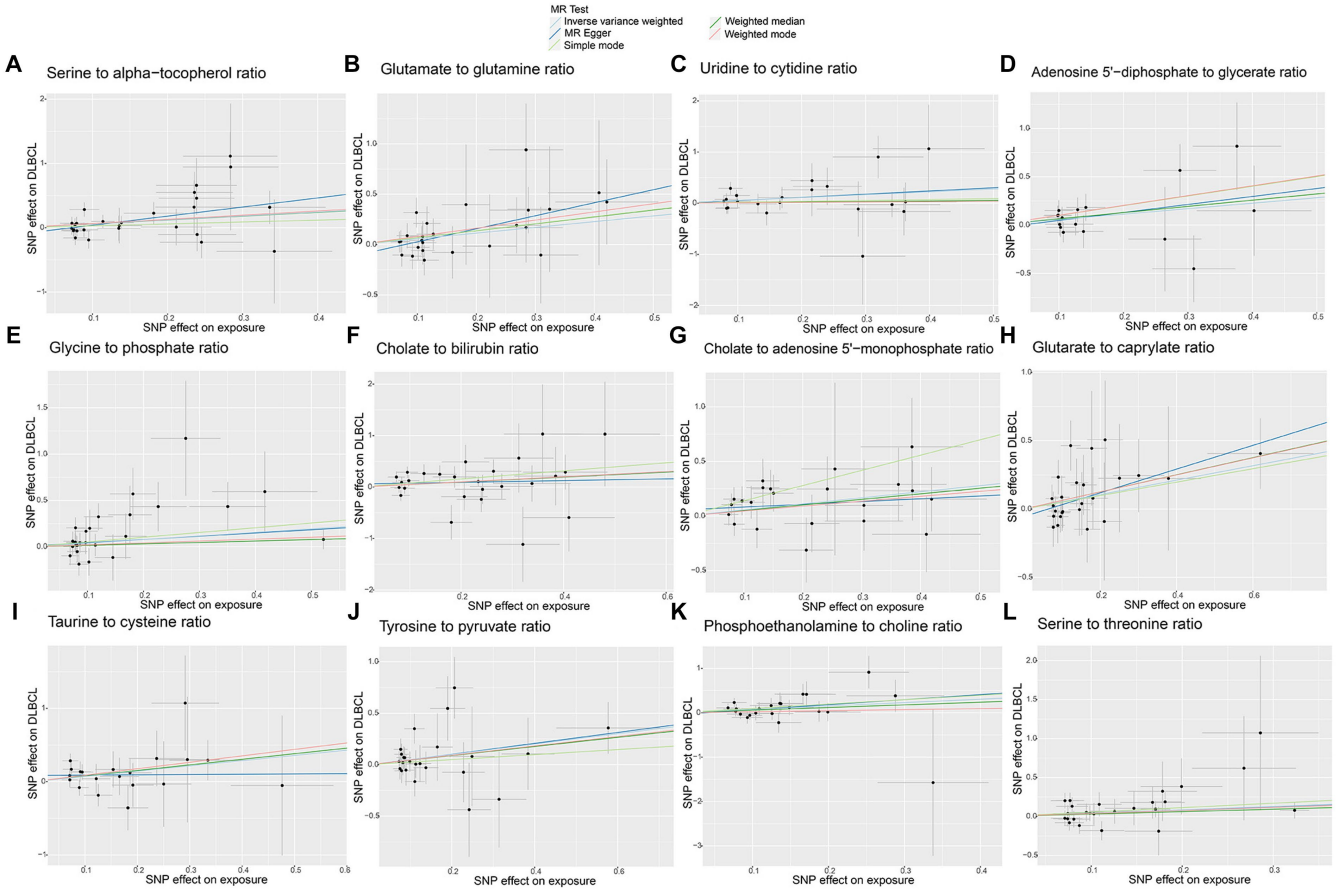
Scatter plots of causal estimates for genetically predicted metabolite ratios contributing to diffuse large B-cell lymphoma (DLBCL) risk. **(A)** Serine to alpha-tocopherol ratio; **(B)** Glutamate to glutamine ratio; **(C)** Uridine to cytidine ratio; **(D)** Adenosine 5’-diphosphate to glycerate ratio; **(E)** Glycine to phosphate ratio; **(F)** Cholate to bilirubin ratio; **(G)** Cholate to adenosine 5’-monophosphate ratio; **(H)** Glutarate to caprylate ratio; **(I)** Taurine to cysteine ratio; **(J)** Tyrosine to pyruvate ratio; **(K)** Phosphoethanolamine to choline ratio; **(L)** Serine to threonine ratio.

No horizontal significant pleiotropy or outliers were detected by MR Egger intercept test, MR-PRESSO test (Supplementary Table S3, *p* > 0.05), or scatter plot (Figures 7, 8). Further, no heterogeneity among the selected SNPs was found by Cochran’s Q-test (Supplementary Table S3, *p* > 0.05) or funnel plot analysis (Supplementary Figures S6, S7). In addition, the stability of MR results was analyzed using leave-one-out plots (Supplementary Figure S8).

## 4 Reverse Mendelian randomization analysis

Reverse MR analysis identified no significant causal influence of DLBCL on the gut microbiota, metabolites, or metabolite ratios described above (Supplementary Tables S4–S6, *p* > 0.05). No significant pleiotropy or heterogeneity was detected by MR Egger intercept test and Cochran’s Q-test (Supplementary Table S7, *p* > 0.05).

## 5 Discussion

Recent, research has identified relationships among gut microbiota, plasma metabolites, and the development of lymphoma (Uribe-Herranz et al., 2021). To our knowledge, this study represents the first MR analysis based on new large-scale GWAS data to identify the causal effects of gut microbiota, plasma metabolites, and metabolites ratios on DLBCL. We report causal relationship of 5 gut microbiota genera, 27 plasma metabolites, and 19 metabolite ratios with DLBCL, providing a reference for potential future interventions and treatments to reduce the risk of DLBCL.

Interactions between the gut flora and the host immune-metabolic system are complex, and can have local and systemic effects on the host (Lozenov et al., 2023; Riazati et al., 2023). Clinical studies or experimental animal studies have demonstrated a relationship between gut microbial composition and disease, and found that dysbiosis appears to be a precursor to carcinogenesis. Using MR analysis, our study is the first to determine that the *Terrisporobacter* and *Oscillibacter* genera represent high-risk flora for DLBCL development, which have potential as specific markers or therapeutic targets. *Terrisporobacter* are anaerobic bacteria, often detected in postoperative patients suffering from comorbidities, such as cirrhosis, abscess, bone infections, and bloodstream infections (Cheng et al., 2016), and are positively associated with sepsis risk (Chen et al., 2023). In addition, invasive fungal disease (IFD) is an important cause of morbidity and mortality in patients with hematologic malignancies. Gavriilaki et al. reported that 19 subjects receiving chimeric antigen receptor T cells and two subjects undergoing gene therapy did not develop IFD, whereas subjects with primary refractory/recurrent lymphoma undergoing autologous hematopoietic cell transplantation (HCT) developed IFD, which was associated with poor outcomes in patients receiving allogeneic HCT (Gavriilaki et al., 2023). Therefore, detection of bacteria or fungi in patients with DLBCL and co-infections warrants attention, to assist in improved patient management. Of interest, there have been reports that intestinal flora may be involved in tumorigenesis and progression through the production of oncogenic exotoxins, oncogenic metabolites, and chronic inflammatory responses. Further, *Oscillibacter* has been reported as closely associated with tumor progression and treatment efficacy. Yu et al. (2023) found that a decrease in the *Oscillibacter* population was associated with reduced GFb and STAT3 expression, and increased levels of TNFa, IFNg, and CXCR4, and that *Oscillibacter* transplantation in conjunction with anticancer immune responses contributed to inhibition of colorectal cancer progression. In addition, Liu et al. (2022) found that increased relative abundance of *Oscillibacter* in feces was correlated with decreased triglyceride levels, while *Oscillibacter* is also reported to be associated with serum metabolite levels related to intestinal flora (Thingholm et al., 2019). Wang et al. (2023) reported that changes in lipid levels in patients with DLBCL were correlated with prognosis and influenced by rituximab efficacy. In addition, preliminary clinical trials demonstrated that the gut microbiota can influence tumor immunotherapy efficacy by enhancing intra-tumoral infiltration of CD8^+^ effector T cells or promoting T cell growth and cytokine production. Xu et al. confirmed that intestinal flora composition differed was significantly between patients with DLBCL and healthy controls, as well as between DLBCL patients before and after treatment with rituximab, cyclophosphamide, doxorubicin, vincristine and prednisone (R-CHOP), and patients in complete and incomplete remission after treatment. Further, intestinal flora composition is correlated with patient immune status and inflammatory factors; in particular, the presence of *Lactobacillus fermentum* during chemotherapy may be associated with better efficacy (Xu et al., 2024). The roles of *Terrisporobacter* or *Oscillibacter* in DLBCL development, and their metabolic and therapeutic impacts, requires further in depth exploration, and additional relevant clinical trials.

Through MR analysis, we also identified a causal association of three microorganisms protective against DLBCL. In response to identification of *Eubacterium coprostanoligenes group* as beneficial, we also found that patients with higher *Eubacterium coprostanoligenes group* abundance exhibited better progression-free survival. In addition, Yuan et al. (2023) used *Eubacterium coprostanoligenes group* and *Prevotella* in construction of a model to estimate the risk of recurrence in patients with hypopharyngeal squamous cell carcinoma, and found that lower abundance of *Eubacterium coprostanoligenes group* was associated with higher recurrence and metastasis rates. *Eubacterium_coprostanoligenes_group* refers to a group of anaerobic Gram-positive bacteria involved in cholesterol transformation and regulation of cholesterol levels. Cholesterol level reduction is reported to inhibit tumor growth and metastasis (Chimento et al., 2018; Huang et al., 2020), while elevated cholesterol levels are correlated with breast cancer recurrence, which can be reduced by the administration of statins. In addition, cholesterol metabolites may promote tumor metastasis by interacting with T cells and neutrophils (Baek et al., 2017). The relationship between *Eubacterium coprostanoligenes group* and cholesterol in DLBCL warrants in depth exploration in the future to provide new insights to inform targeted therapy.

In recent years, infection with a number of agents, such as Epstein–Barr virus (EBV), human herpesvirus 8, and human immunodeficiency virus infection, has been strongly associated with the risk of developing NHL. Identifying possible pathogens correlated with NHL and understanding the relationship between NHL and pathogens is crucial for disease prevention and screening. Siqueira et al. (2023) showed that there is viral diversity in NHL. Joo et al. (2021) found that *Eubacterium coprostanoligenes* was significantly increased in patients with low HBV DNA, suggesting a relationship between gut flora composition and chronic HBV infection load. More importantly, HIV-infected patients have been identified as at increased risk for hematologic neoplasms, of which DLBCL is the most common type. Although little is known about the pathogenesis of HIV-associated DLBCL, Huguet et al. (2023) reported an improved rate of complete remission in patients treated with conventional chemotherapy combined with antiretroviral therapy. Direct or indirect interactions between intestinal bacteria and the intestinal mucosal immune system can modulate physiological immune response. *Slackia* has been reported as potentially related to adaptive immune activation, as it is positively correlating with IF13 production, as well as the T-cell cytokines, IL-10, IFN-γ, and IL-17, which contribute to memory T cells activations (Margiotta et al., 2021). Regarding *Methanobrevibacter*, there are reports that adjuvants can overcome tolerance to tumor-associated melanoma antigens and induce CD8+ T cell responses (Krishnan et al., 2010). Together, these studies suggest that focusing on the management and modification of patient intestinal flora during consultations with clinicians may help to reduce the risk of DLBCL development and improve patient outcomes.

Changes in metabolism lead to metabolic phenotypes, which can serve as biomarkers for early detection of cancer and treatment optimization (Luengo et al., 2017). There is an urgent need for identification of metabolites that can be assessed using non-invasive body fluid samples (such as blood, urine, etc.) as biomarkers to help diagnose lymphoma. Hexokinase 2 (HK2) is an important regulator involved in glucose metabolism, and is associated with carcinogenesis in various malignant tumors. Zhao et al. reported that HK2 exerts a malignant biological effect on DLBCL cells through ERK1/2 signaling (Zhao et al., 2023). In this study, we detected causal relationships of plasma metabolites and metabolite ratios with DLBCL, particularly the metabolism of amino acids. Some hematological tumors are reported to exhibit high asparagines consumption rates, which maintains malignant tumor cell growth. Asparagine is associated with mTORC1 activity and can regulate the uptake of amino acids, such as serine. Many tumor cells rely heavily on serine to support a functional nucleotide library, which facilitates cell proliferation (Eraslan et al., 2021). Our data also indicate that serine/threonine and serine/α-tocopherol ratios are causally related to high risk of DLBCL. Fouad Choueiry et al. conducted a metabolomics and gene expression study and found that alanine, cysteine, aspartic acid, glutamic acid, and methionine metabolism were all dysregulated in ibrutinib-resistant activated B cell-DLBCL (Choueiry et al., 2021). Our study also revealed that 4-hydroxyglutamate levels, glutamate/glutamine ratio, glutarate (C5-DC)/caprylate (8:0) ratio, and taurine/cysteine ratio were associated with high risk of DLBCL. Additionally, we identified a causal effect of phosphoethanolamine/choline ratio on DLBCL risk. Xiong et al. (2017) identified a direct correlation between MYC overexpression and dysregulation of choline metabolism, and reported that MYC disrupts choline metabolism and hinders lymphoma cell necroptosis in a mitochondrial autophagy-dependent manner, by activating phosphohistidine transferase 1 choline-α. Further study is needed to explore the role and clinical value of metabolites in DLBCL occurrence and progression.

Our research has multiple strengths. First, our study was the first to apply MR analysis to investigate the causal effects of gut microbiota, plasma metabolites, and metabolite ratios in DLBCL. Compared with traditional retrospective clinical studies, MR analysis is more reliable, because it reduces bias caused by confounding factors. The candidate gut bacteria and plasma metabolites identified in this study provide a foundation for subsequent research into the underlying mechanisms, which could help to discover novel diagnostic biomarkers and personalized treatment strategies for patients with DLBCL. Second, SNPs related to gut microbiota and metabolites were sourced from a large GWAS dataset, ensuring the reliability of the screened IVs. Additionally, the statistical processing capability of R software and corresponding sensitivity analyses reduced the effects of bias on our results, ensuring their stability and reliability. Nevertheless, this study has some limitations. Most subjects included in the GWAS were of European ethnicity, which may led to some bias. Further, the minimum classification level included in the gut microbiota dataset was genus, preventing investigation into causal correlations at the species level. In addition, we were unable to perform subgroup analysis, for example, by stratifying germinal center B-cell like and activated B-cell like disease subtypes. Further research is needed to elucidate the relationships of gut microbiota, plasma metabolites, and metabolite ratios with DLBCL, and to explore the role of gut microbiota and metabolites on the gut barrier, host immune responses, and homeostasis.

## 6 Conclusion

In summary, our study applied MR analysis to determine the causal effects of 5 gut microbiota, 27 plasma metabolites, and 19 metabolite ratios on DLBCL. Our research findings have potential to provide new directions to inform the prevention, auxiliary diagnosis, and treatment cure of DLBCL, by targeting gut microbiota or metabolites. Further research to determine the underlying mechanisms involved is warranted.

## Data availability statement

Publicly available datasets were analyzed in this study. This data can be found here: gut microbiota: https://mibiogen.gcc.rug.nl/; diffuse large B-cell lymphoma: https://gwas.mrcieu.ac.uk/. The original contributions presented in the study are included in the article and supplementary material, further inquiries can be directed to the corresponding authors.

## Ethics statement

Ethical approval was not required for the study involving humans in accordance with the local legislation and institutional requirements. Written informed consent to participate in this study was not required from the participants or the participants’ legal guardians/next of kin in accordance with the national legislation and the institutional requirements.

## Author contributions

JQ: Data curation, Project administration, Writing – original draft, Writing – review & editing. WZ: Methodology, Software, Writing – original draft. JF: Data curation, Formal analysis, Visualization, Writing – original draft. SC: Investigation, Supervision, Validation, Writing – review & editing. YZ: Investigation, Resources, Visualization, Writing – review & editing. XZ: Project administration, Writing – review & editing. CS: Conceptualization, Project administration, Writing – review & editing.

## Footnotes

^1^https://gwas.mrcieu.ac.uk/, Updated to November 2023.

## Supplementary material

SUPPLEMENTARY FIGURE S1 Funnel plots of causal estimates for genetically predicted gut microbiota on diffuse large B-cell lymphoma (DLBCL) risk.

SUPPLEMENTARY FIGURE S2 Leave-one-out plots of causal estimates for genetically predicted microbiota on diffuse large B-cell lymphoma (DLBCL) risk.

SUPPLEMENTARY FIGURE S3 Funnel plots of causal estimates for genetically predicted protective plasma metabolites on diffuse large B-cell lymphoma (DLBCL) risk.

SUPPLEMENTARY FIGURE S4 Funnel plots of causal estimates for genetically predicted plasma metabolites contributing to diffuse large B-cell lymphoma (DLBCL) risk.

SUPPLEMENTARY FIGURE S5 Leave-one-out plots of causal estimates for genetically predicted plasma metabolites on diffuse large B-cell lymphoma (DLBCL) risk.

SUPPLEMENTARY FIGURE S6 Funnel plots of causal estimates for genetically predicted protective metabolite ratios on diffuse large B-cell lymphoma (DLBCL) risk.

SUPPLEMENTARY FIGURE S7 Funnel plots of causal estimates for genetically predicted metabolite ratios contributing to diffuse large B-cell lymphoma (DLBCL) risk.

SUPPLEMENTARY FIGURE S8 Leave-one-out plots of causal estimates for genetically predicted metabolite ratios on diffuse large B-cell lymphoma (DLBCL) risk.
