# Supplementary figures and images for: Causal relationships of gut microbiota, plasma metabolites, and metabolite ratios with diffuse large B-cell lymphoma: a Mendelian randomization study
Source: Front Microbiol. 2024 May 27;15:1356437. doi: 10.3389/fmicb.2024.1356437 (PMC11163048; doi:10.3389/fmicb.2024.1356437)

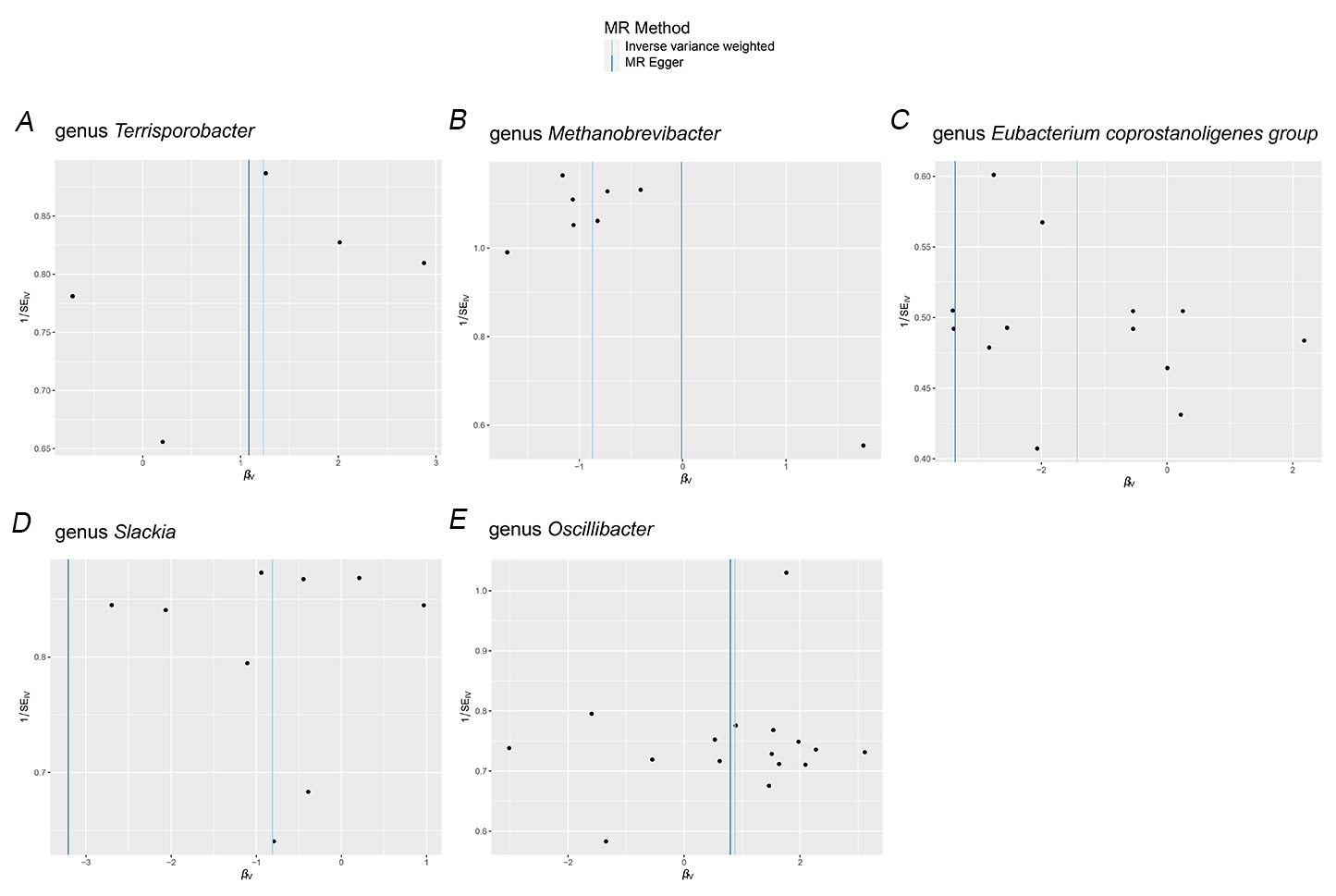

Supplement: SUPPLEMENTARY FIGURE S1 — Funnel plots of causal estimates for genetically predicted gut microbiota on diffuse large B-cell lymphoma (DLBCL) risk. [file Image_1.jpeg]

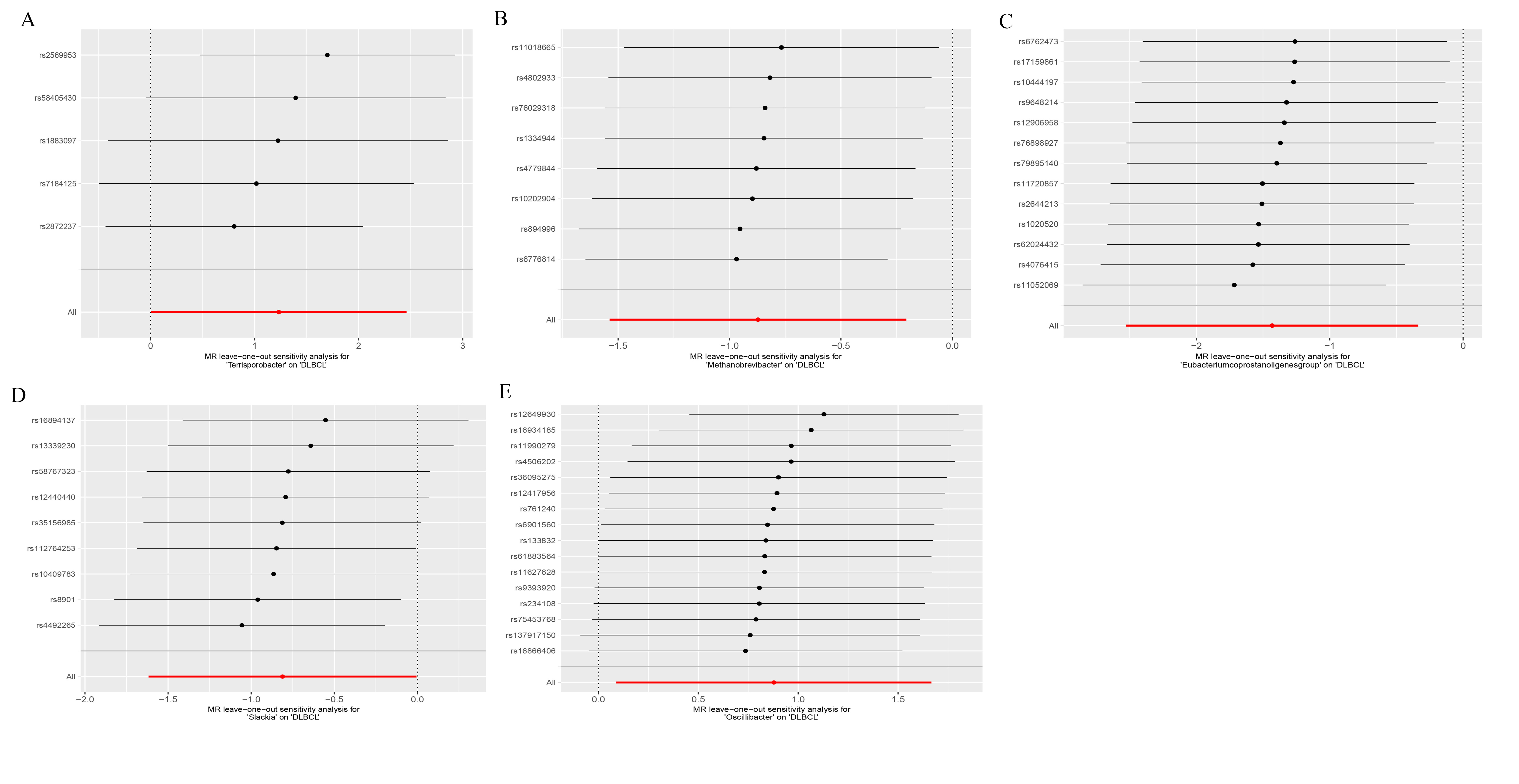

Supplement: SUPPLEMENTARY FIGURE S2 — Leave-one-out plots of causal estimates for genetically predicted microbiota on diffuse large B-cell lymphoma (DLBCL) risk. [file Image_2.jpeg]

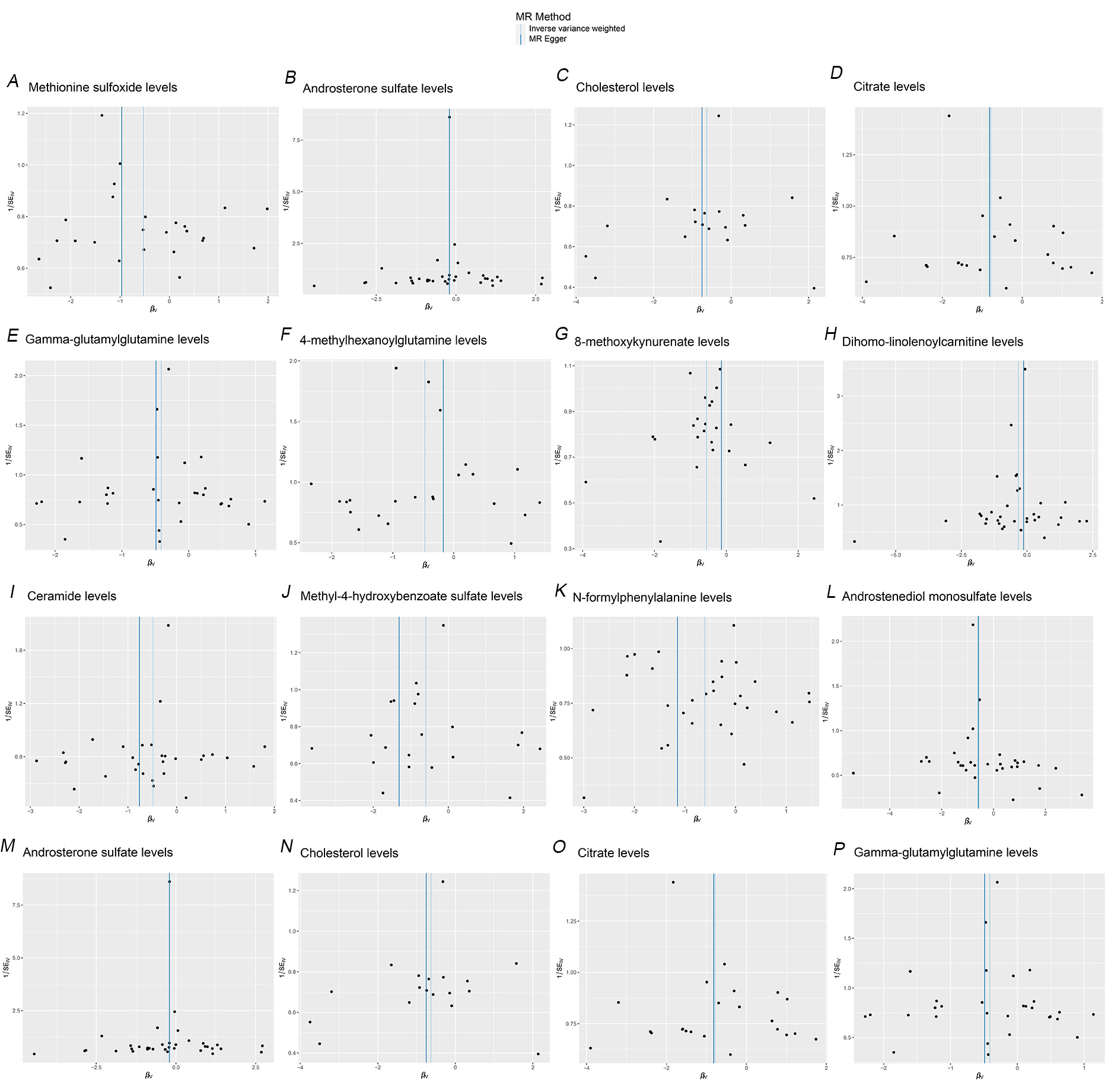

Supplement: SUPPLEMENTARY FIGURE S3 — Funnel plots of causal estimates for genetically predicted protective plasma metabolites on diffuse large B-cell lymphoma (DLBCL) risk. [file Image_3.jpeg]

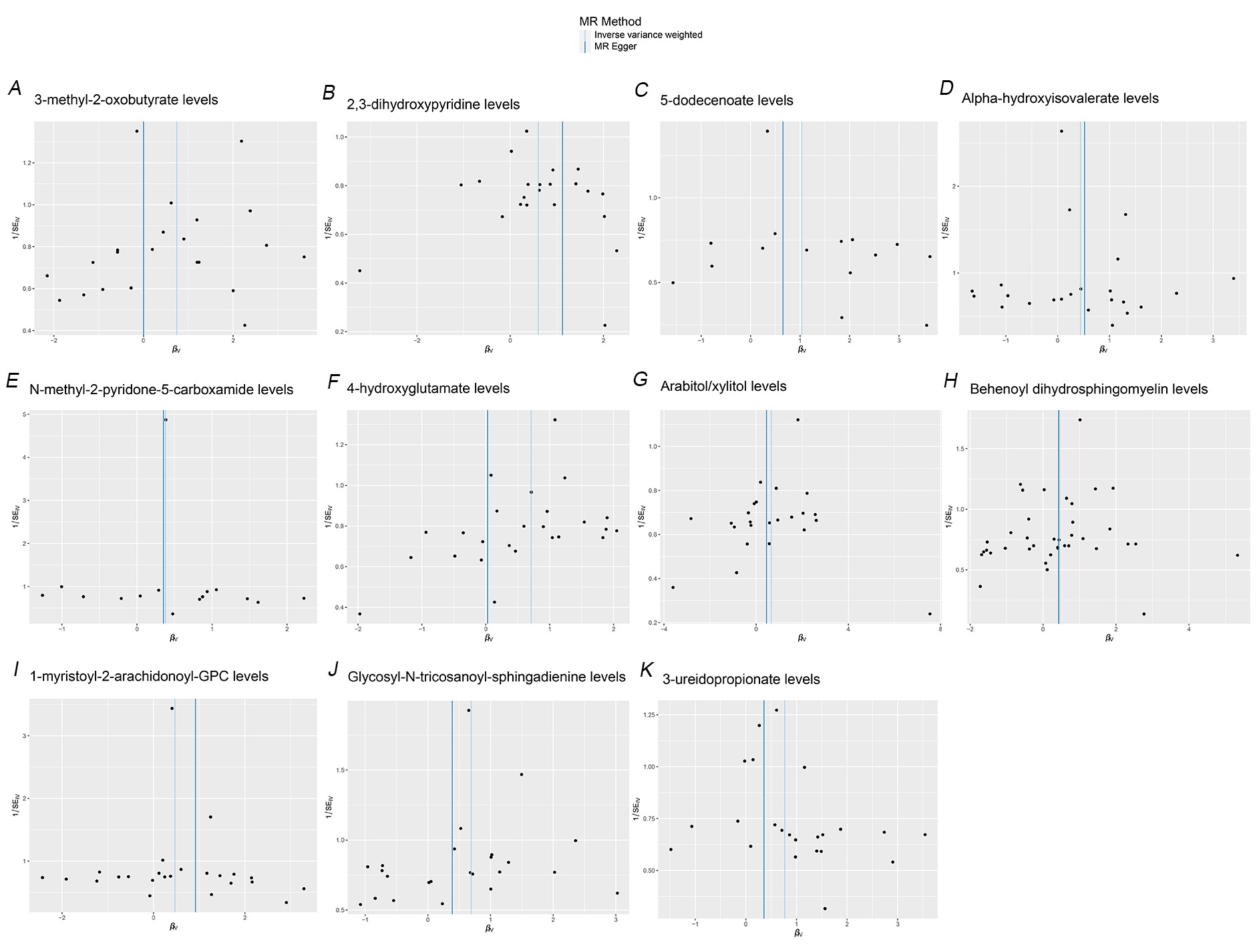

Supplement: SUPPLEMENTARY FIGURE S4 — Funnel plots of causal estimates for genetically predicted plasma metabolites contributing to diffuse large B-cell lymphoma (DLBCL) risk. [file Image_4.jpeg]

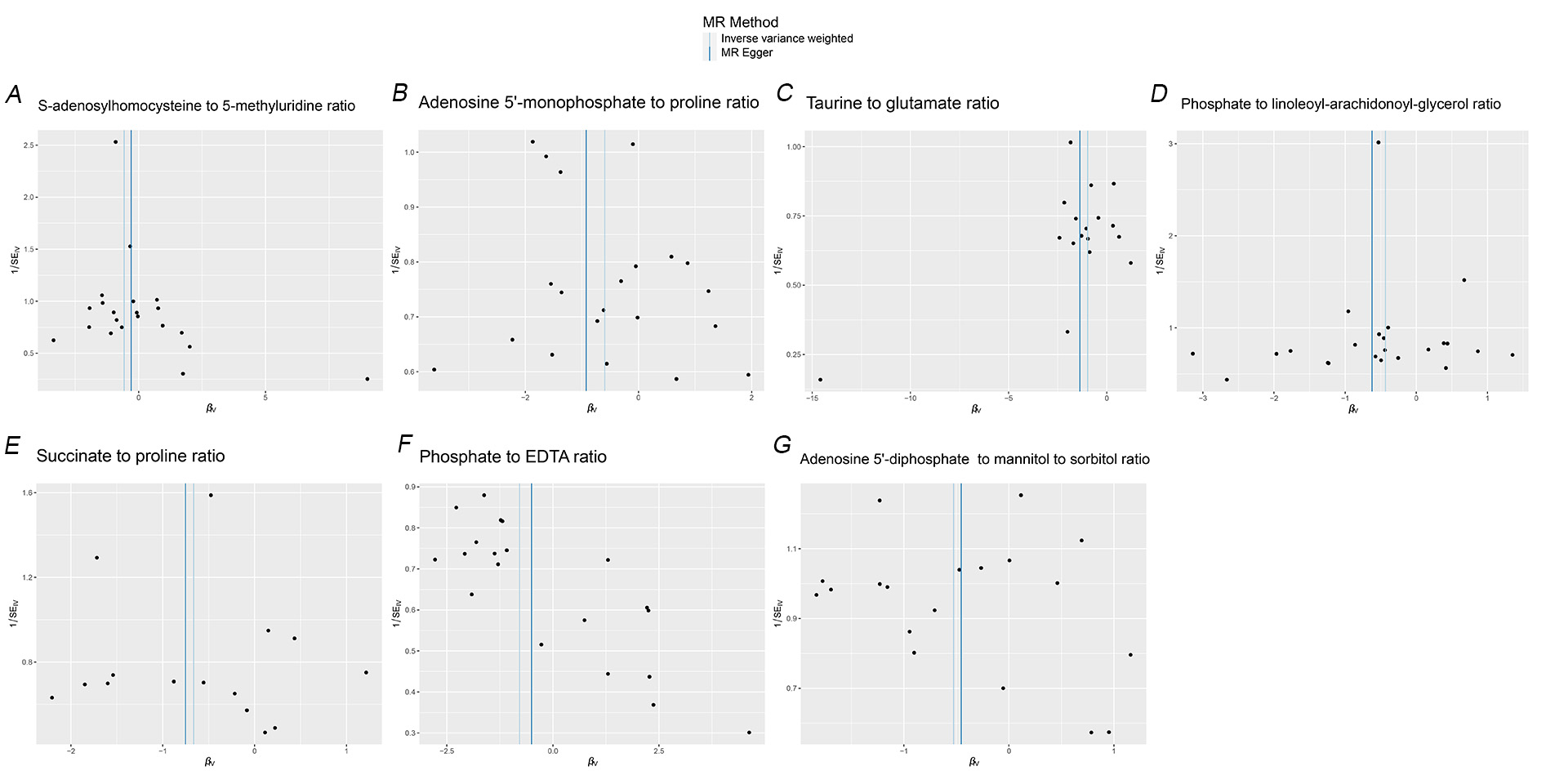

Supplement: SUPPLEMENTARY FIGURE S6 — Funnel plots of causal estimates for genetically predicted protective metabolite ratios on diffuse large B-cell lymphoma (DLBCL) risk. [file Image_6.jpeg]

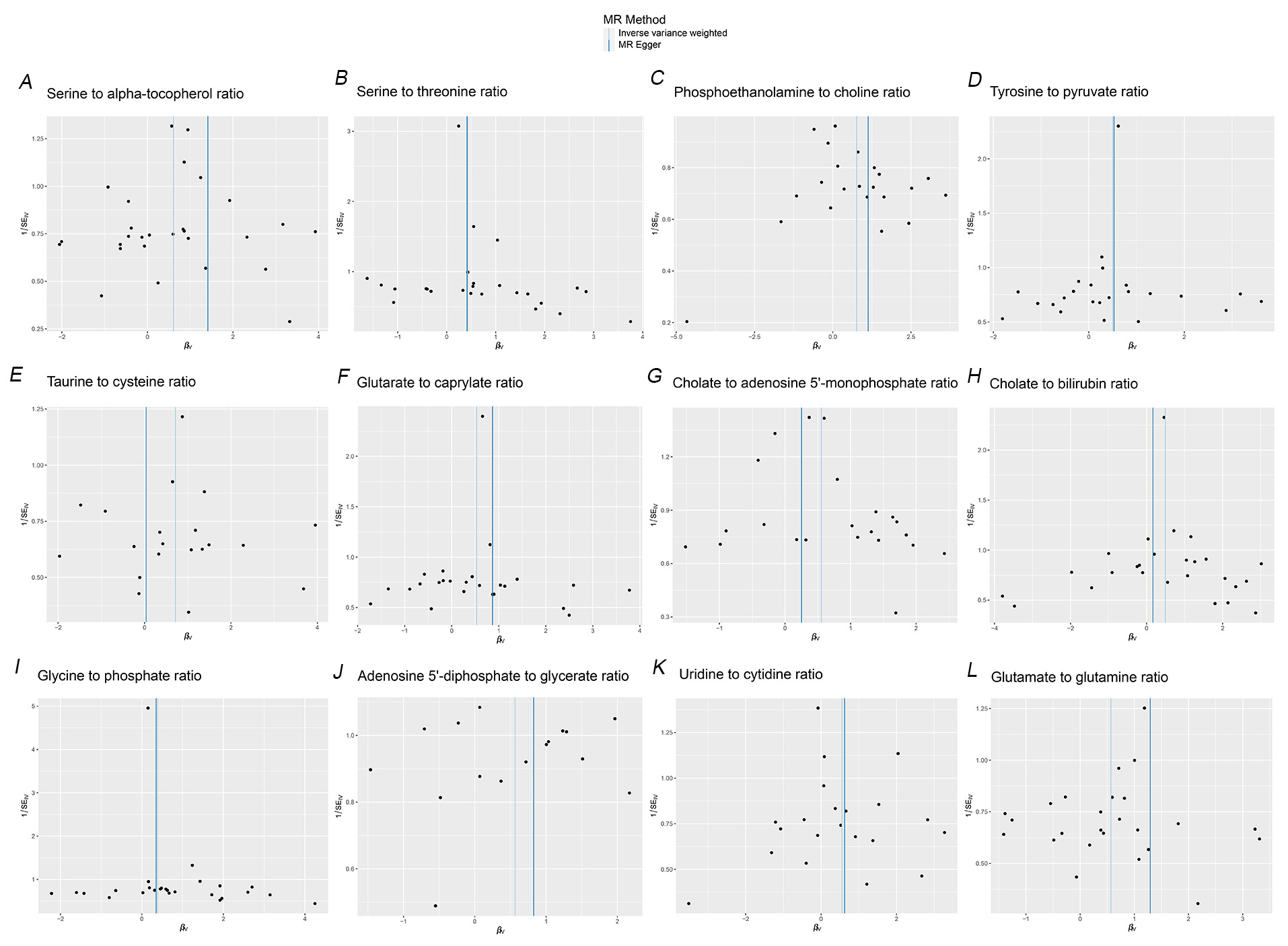

Supplement: SUPPLEMENTARY FIGURE S7 — Funnel plots of causal estimates for genetically predicted metabolite ratios contributing to diffuse large B-cell lymphoma (DLBCL) risk. [file Image_7.jpeg]
