# Supplementary material for: Causal relationships of gut microbiota, plasma metabolites, and metabolite ratios with diffuse large B-cell lymphoma: a Mendelian randomization study
Source: Front Microbiol. 2024 May 27;15:1356437. doi: 10.3389/fmicb.2024.1356437 (PMC11163048; doi:10.3389/fmicb.2024.1356437)
Supplement: Supplementary file 9 [file Table_1.docx]

Supplement Table S1. Mendelian randomized pleiotropy and heterogeneity test of gut microbiota and Diffuse large B-cell lymphoma.

| **Exposure** | **Outcome** | **MR-Egger-intercept**  ***P-*value** | **MR-PRESSO Global -test**  ***P-*value** | **Cochran’s Q**  ***P-*value** |
| --- | --- | --- | --- | --- |
| Terrisporobacter | DLBCL | 0.947 | 0.339 | 0.175 (MR Egger) |
|  |  |  |  | 0.291 (IVW) |
| Methanobrevibacter | DLBCL | 0.532 | 0.900 | 0.829 (MR Egger) |
|  |  |  |  | 0.858 (IVW) |
| Eubacterium coprostanoligenes group | DLBCL | 0.380 | 0.752 | 0.735 (MR Egger) |
|  |  |  |  | 0.737 (IVW) |
| Slackia | DLBCL | 0.240 | 0.556 | 0.624 (MR Egger) |
|  |  |  |  | 0.542 (IVW) |
| Oscillibacter | DLBCL | 0.954 | 0.115 | 0.081 (MR Egger) |
|  |  |  |  | 0.111 (IVW) |

Supplement Table S2. Mendelian randomized pleiotropy and heterogeneity test of plasma metabolites and Diffuse large B-cell lymphoma.

| **Exposure** | **Outcome** | **MR-Egger-intercept *P-*value** | **MR-PRESSO Global -test**  ***P-*value** | **Cochran’s Q**  ***P-*value** |
| --- | --- | --- | --- | --- |
| Methionine sulfoxide levels | DLBCL | 0.384 | 0.667 | 0.642 (MR Egger) |
|  |  |  |  | 0.651 (IVW) |
| DHEAS levels | DLBCL | 0.779 | 0.258 | 0.186 (MR Egger) |
|  |  |  |  | 0.213 (IVW) |
| 3-methyl-2-oxobutyrate levels | DLBCL | 0.267 | 0.123 | 0.136 (MR Egger) |
|  |  |  |  | 0.121 (IVW) |
| 2,3-dihydroxypyridine levels | DLBCL | 0.586 | 0.965 | 0.943 (MR Egger) |
|  |  |  |  | 0.954 (IVW) |
| Glycolithocholate levels | DLBCL | 0.777 | 0.751 | 0.697 (MR Egger) |
|  |  |  |  | 0.745 (IVW) |
| 5-dodecenoate levels | DLBCL | 0.593 | 0.51 | 0.428 (MR Egger) |
|  |  |  |  | 0.482 (IVW) |
| Alpha-hydroxyisovalerate levels | DLBCL | 0.835 | 0.338 | 0.280 (MR Egger) |
|  |  |  |  | 0.331 (IVW) |
| N-methyl-2-pyridone-5-carboxamide levels | DLBCL | 0.852 | 0.91 | 0.815 (MR Egger) |
|  |  |  |  | 0.864 (IVW) |
| 4-ethylphenylsulfate levels | DLBCL | 0.888 | 0.632 | 0.564 (MR Egger) |
|  |  |  |  | 0.623 (IVW) |
| 5alpha-pregnan-3beta,20alpha-diol monosulfate levels | DLBCL | 0.792 | 0.861 | 0.796 (MR Egger) |
|  |  |  |  | 0.832 (IVW) |
| Androstenediol monosulfate levels | DLBCL | 0.935 | 0.734 | 0.661 (MR Egger) |
|  |  |  |  | 0.707 (IVW) |
| 4-hydroxyglutamate levels | DLBCL | 0.150 | 0.986 | 0.995 (MR Egger) |
|  |  |  |  | 0.985 (IVW) |
| N-formylphenylalanine levels | DLBCL | 0.281 | 0.865 | 0.876 (MR Egger) |
|  |  |  |  | 0.864 (IVW) |
| Methyl-4-hydroxybenzoate sulfate levels | DLBCL | 0.176 | 0.393 | 0.420 (MR Egger) |
|  |  |  |  | 0.361 (IVW) |
| Arabitol/xylitol levels | DLBCL | 0.776 | 0.393 | 0.343 (MR Egger) |
|  |  |  |  | 0.394 (IVW) |
| Behenoyl dihydrosphingomyelin levels | DLBCL | 0.952 | 0.456 | 0.394 (MR Egger) |
|  |  |  |  | 0.439 (IVW) |
| 1-myristoyl-2-arachidonoyl-GPC levels | DLBCL | 0.112 | 0.527 | 0.575 (MR Egger) |
|  |  |  |  | 0.468 (IVW) |
| Glycosyl-N-tricosanoyl-sphingadienine levels | DLBCL | 0.519 | 0.863 | 0.836 (MR Egger) |
|  |  |  |  | 0.855 (IVW) |
| Ceramide levels | DLBCL | 0.548 | 0.778 | 0.742 (MR Egger) |
|  |  |  |  | 0.768 (IVW) |
| Dihomo-linolenoylcarnitine levels | DLBCL | 0.364 | 0.587 | 0.553 (MR Egger) |
|  |  |  |  | 0.560 (IVW) |
| 8-methoxykynurenate levels | DLBCL | 0.500 | 0.962 | 0.949 (MR Egger) |
|  |  |  |  | 0.955 (IVW) |
| 4-methylhexanoylglutamine levels | DLBCL | 0.372 | 0.635 | 0.594 (MR Egger) |
|  |  |  |  | 0.602 (IVW) |
| 3-ureidopropionate levels | DLBCL | 0.384 | 0.91 | 0.904 (MR Egger) |
|  |  |  |  | 0.904 (IVW) |
| Gamma-glutamylglutamine levels | DLBCL | 0.828 | 0.991 | 0.991 (MR Egger) |
|  |  |  |  | 0.994 (IVW) |
| Citrate levels | DLBCL | 0.963 | 0.18 | 0.137 (MR Egger) |
|  |  |  |  | 0.172 (IVW) |
| Cholesterol levels | DLBCL | 0.831 | 0.628 | 0.537 (MR Egger) |
|  |  |  |  | 0.604 (IVW) |
| Androsterone sulfate levels | DLBCL | 0.956 | 0.455 | 0.271 (MR Egger) |
|  |  |  |  | 0.312 (IVW) |

Supplement Table S3. Mendelian randomized pleiotropy and heterogeneity test of metabolite ratios and Diffuse large B-cell lymphoma.

| **Exposure** | **Outcome** | **MR-Egger-intercept**  ***P-*value** | **MR-PRESSO Global -test**  ***P-*value** | **Cochran’s Q**  ***P-*value** |
| --- | --- | --- | --- | --- |
| S-adenosylhomocysteine to 5-methyluridine ratio | DLBCL | 0.586 | 0.263 | 0.189 (MR Egger) |
|  |  |  |  | 0.218 (IVW) |
| Adenosine 5'-monophosphate to proline ratio | DLBCL | 0.553 | 0.563 | 0.521 (MR Egger) |
|  |  |  |  | 0.562 (IVW) |
| Serine to alpha-tocopherol ratio | DLBCL | 0.112 | 0.313 | 0.368 (MR Egger) |
|  |  |  |  | 0.283 (IVW) |
| Glutamate to glutamine ratio | DLBCL | 0.178 | 0.883 | 0.932 (MR Egger) |
|  |  |  |  | 0.897 (IVW) |
| Uridine to cytidine ratio | DLBCL | 0.89 | 0.472 | 0.396 (MR Egger) |
|  |  |  |  | 0.457 (IVW) |
| Adenosine 5'-diphosphate to glycerate ratio | DLBCL | 0.722 | 0.574 | 0.513 (MR Egger) |
|  |  |  |  | 0.579 (IVW) |
| Glycine to phosphate ratio | DLBCL | 0.843 | 0.322 | 0.279 (MR Egger) |
|  |  |  |  | 0.326 (IVW) |
| Cholate to bilirubin ratio | DLBCL | 0.4558 | 0.238 | 0.15 (MR Egger) |
|  |  |  |  | 0.162 (IVW) |
| Cholate to adenosine 5'-monophosphate ratio | DLBCL | 0.41 | 0.856 | 0.84 (MR Egger) |
|  |  |  |  | 0.846 (IVW) |
| Taurine to glutamate ratio | DLBCL | 0.62 | 0.728 | 0.621 (MR Egger) |
|  |  |  |  | 0.672 (IVW) |
| Glutarate to caprylate (8:0) ratio | DLBCL | 0.335 | 0.895 | 0.864 (MR Egger) |
|  |  |  |  | 0.859 (IVW) |
| Taurine to cysteine ratio | DLBCL | 0.291 | 0.555 | 0.558 (MR Egger) |
|  |  |  |  | 0.544 (IVW) |
| Phosphate to linoleoyl-arachidonoyl-glycerol ratio | DLBCL | 0.605 | 0.871 | 0.843 (MR Egger) |
|  |  |  |  | 0.868 (IVW) |
| Tyrosine to pyruvate ratio | DLBCL | 0.939 | 0.673 | 0.564 (MR Egger) |
|  |  |  |  | 0.623 (IVW) |
| Succinate to proline ratio | DLBCL | 0.872 | 0.841 | 0.808 (MR Egger) |
|  |  |  |  | 0.859 (IVW) |
| Phosphate to EDTA ratio | DLBCL | 0.81 | 0.262 | 0.184 (MR Egger) |
|  |  |  |  | 0.227 (IVW) |
| Adenosine 5'-diphosphate to mannitol to sorbitol ratio | DLBCL | 0.89 | 0.784 | 0.705 (MR Egger) |
|  |  |  |  | 0.763 (IVW) |
| Phosphoethanolamine to choline ratio | DLBCL | 0.641 | 0.543 | 0.482 (MR Egger) |
|  |  |  |  | 0.531 (IVW) |
| Serine to threonine ratio | DLBCL | 0.97 | 0.762 | 0.658 (MR Egger) |
|  |  |  |  | 0.712 (IVW) |

Supplement Table S4. Reverse Mendelian randomized analysis for the relation between gut microbiota and Diffuse large B-cell lymphoma.

| **Exposure** | **Outcome** | **MR method** | **β** | **SE** | **OR** | **95% CI** | | ***P*-value** |
| --- | --- | --- | --- | --- | --- | --- | --- | --- |
| DLBCL | Terrisporobacter | MR Egger | -0.010 | 0.043 | 0.990 | 0.911 | 1.077 | 0.820 |
|  |  | Weighted median | 0.015 | 0.015 | 1.015 | 0.986 | 1.045 | 0.324 |
|  |  | Inverse variance weighted | 0.019 | 0.011 | 1.019 | 0.997 | 1.041 | 0.089 |
|  |  | Weighted mode | 0.010 | 0.023 | 1.011 | 0.965 | 1.058 | 0.664 |
|  |  | Simple mode | 0.012 | 0.024 | 1.012 | 0.966 | 1.059 | 0.634 |
| DLBCL | Methanobrevibacter | MR Egger | 0.021 | 0.067 | 1.021 | 0.895 | 1.166 | 0.762 |
|  |  | Weighted median | 0.021 | 0.022 | 1.022 | 0.979 | 1.066 | 0.323 |
|  |  | Inverse variance weighted | 0.016 | 0.015 | 1.016 | 0.986 | 1.048 | 0.295 |
|  |  | Weighted mode | 0.024 | 0.033 | 1.024 | 0.960 | 1.092 | 0.486 |
|  |  | Simple mode | 0.022 | 0.033 | 1.022 | 0.958 | 1.092 | 0.525 |
| DLBCL | Eubacterium coprostanoligenes group | MR Egger | 0.003 | 0.027 | 1.003 | 0.951 | 1.058 | 0.908 |
|  |  | Weighted median | -0.010 | 0.009 | 0.990 | 0.973 | 1.008 | 0.277 |
|  |  | Inverse variance weighted | -0.005 | 0.007 | 0.995 | 0.982 | 1.008 | 0.471 |
|  |  | Weighted mode | -0.019 | 0.016 | 0.981 | 0.951 | 1.012 | 0.253 |
|  |  | Simple mode | -0.018 | 0.016 | 0.982 | 0.951 | 1.014 | 0.285 |
| DLBCL | Slackia | MR Egger | -0.035 | 0.044 | 0.965 | 0.886 | 1.052 | 0.443 |
|  |  | Weighted median | -0.007 | 0.015 | 0.993 | 0.965 | 1.022 | 0.630 |
|  |  | Inverse variance weighted | -0.011 | 0.011 | 0.989 | 0.968 | 1.012 | 0.349 |
|  |  | Weighted mode | -0.008 | 0.024 | 0.992 | 0.947 | 1.040 | 0.742 |
|  |  | Simple mode | -0.006 | 0.026 | 0.994 | 0.945 | 1.046 | 0.822 |
| DLBCL | Oscillibacter | MR Egger | -0.019 | 0.036 | 0.981 | 0.914 | 1.054 | 0.619 |
|  |  | Weighted median | -0.007 | 0.013 | 0.993 | 0.969 | 1.018 | 0.578 |
|  |  | Inverse variance weighted | -0.004 | 0.009 | 0.996 | 0.977 | 1.014 | 0.642 |
|  |  | Weighted mode | -0.005 | 0.021 | 0.995 | 0.955 | 1.037 | 0.811 |
|  |  | Simple mode | -0.005 | 0.019 | 0.995 | 0.958 | 1.034 | 0.806 |

Abbreviation: MR,Mendelian randomization; SNP,single nucleotide polymorphisms; β, Beta; SE,standard error; OR, odds ratio; CI, confidence interval.

Supplement Table S5. Reverse Mendelian randomized analysis for the relation between plasma metabolites and Diffuse large B-cell lymphoma.

| **Exposure** | **Outcome** | **MR method** | **β** | **SE** | **OR** | **95% CI** | | ***P*-value** |
| --- | --- | --- | --- | --- | --- | --- | --- | --- |
| DLBCL | Methionine sulfoxide levels | MR Egger | -0.007 | 0.014 | 0.993 | 0.966 | 1.021 | 0.625 |
|  |  | Weighted median | 0.001 | 0.010 | 1.001 | 0.981 | 1.021 | 0.935 |
|  |  | Inverse variance weighted | -0.006 | 0.007 | 0.994 | 0.980 | 1.008 | 0.415 |
|  |  | Weighted mode | 0.008 | 0.015 | 1.008 | 0.979 | 1.038 | 0.610 |
|  |  | Simple mode | 0.010 | 0.017 | 1.010 | 0.977 | 1.044 | 0.567 |
| DLBCL | DHEAS levels | MR Egger | -0.019 | 0.012 | 0.981 | 0.958 | 1.005 | 0.136 |
|  |  | Weighted median | -0.004 | 0.009 | 0.996 | 0.978 | 1.014 | 0.636 |
|  |  | Inverse variance weighted | 0.009 | 0.007 | 1.009 | 0.996 | 1.022 | 0.168 |
|  |  | Weighted mode | -0.008 | 0.013 | 0.992 | 0.968 | 1.017 | 0.554 |
|  |  | Simple mode | -0.004 | 0.015 | 0.996 | 0.967 | 1.026 | 0.796 |
| DLBCL | 3-methyl-2-oxobutyrate levels | MR Egger | -0.008 | 0.014 | 0.992 | 0.965 | 1.020 | 0.590 |
|  |  | Weighted median | -0.007 | 0.010 | 0.993 | 0.974 | 1.013 | 0.487 |
|  |  | Inverse variance weighted | 0.002 | 0.007 | 1.002 | 0.988 | 1.016 | 0.797 |
|  |  | Weighted mode | -0.008 | 0.013 | 0.992 | 0.967 | 1.017 | 0.541 |
|  |  | Simple mode | -0.011 | 0.015 | 0.989 | 0.960 | 1.019 | 0.473 |
| DLBCL | 2,3-dihydroxypyridine levels | MR Egger | 0.017 | 0.015 | 1.017 | 0.986 | 1.048 | 0.302 |
|  |  | Weighted median | 0.004 | 0.011 | 1.004 | 0.982 | 1.025 | 0.738 |
|  |  | Inverse variance weighted | 0.006 | 0.008 | 1.006 | 0.990 | 1.022 | 0.464 |
|  |  | Weighted mode | 0.005 | 0.016 | 1.005 | 0.974 | 1.037 | 0.756 |
|  |  | Simple mode | -0.002 | 0.018 | 0.998 | 0.963 | 1.035 | 0.936 |
| DLBCL | Glycolithocholate levels | MR Egger | 0.017 | 0.015 | 1.017 | 0.987 | 1.048 | 0.282 |
|  |  | Weighted median | 0.012 | 0.010 | 1.012 | 0.992 | 1.033 | 0.238 |
|  |  | Inverse variance weighted | 0.017 | 0.008 | 1.017 | 1.002 | 1.033 | 0.027 |
|  |  | Weighted mode | 0.011 | 0.020 | 1.011 | 0.972 | 1.051 | 0.581 |
|  |  | Simple mode | 0.012 | 0.020 | 1.012 | 0.973 | 1.052 | 0.554 |
| DLBCL | 5-dodecenoate levels | MR Egger | 0.006 | 0.013 | 1.006 | 0.981 | 1.031 | 0.668 |
|  |  | Weighted median | 0.005 | 0.009 | 1.006 | 0.988 | 1.024 | 0.545 |
|  |  | Inverse variance weighted | 0.002 | 0.007 | 1.002 | 0.989 | 1.015 | 0.767 |
|  |  | Weighted mode | 0.006 | 0.012 | 1.006 | 0.983 | 1.030 | 0.601 |
|  |  | Simple mode | 0.004 | 0.015 | 1.004 | 0.974 | 1.034 | 0.816 |
| DLBCL | Alpha-hydroxyisovalerate levels | MR Egger | -0.010 | 0.013 | 0.990 | 0.965 | 1.016 | 0.465 |
|  |  | Weighted median | 0.007 | 0.010 | 1.007 | 0.989 | 1.026 | 0.450 |
|  |  | Inverse variance weighted | 0.004 | 0.007 | 1.004 | 0.991 | 1.018 | 0.556 |
|  |  | Weighted mode | 0.016 | 0.015 | 1.016 | 0.986 | 1.046 | 0.308 |
|  |  | Simple mode | 0.022 | 0.018 | 1.023 | 0.987 | 1.059 | 0.233 |
| DLBCL | N-methyl-2-pyridone-5-carboxamide levels | MR Egger | 0.034 | 0.014 | 1.035 | 1.007 | 1.063 | 0.028 |
|  |  | Weighted median | 0.002 | 0.011 | 1.002 | 0.981 | 1.023 | 0.849 |
|  |  | Inverse variance weighted | -0.002 | 0.008 | 0.998 | 0.982 | 1.014 | 0.796 |
|  |  | Weighted mode | -0.019 | 0.021 | 0.981 | 0.941 | 1.023 | 0.379 |
|  |  | Simple mode | -0.026 | 0.021 | 0.974 | 0.934 | 1.016 | 0.238 |
| DLBCL | 4-ethylphenylsulfate levels | MR Egger | -0.002 | 0.014 | 0.998 | 0.971 | 1.026 | 0.882 |
|  |  | Weighted median | 0.004 | 0.010 | 1.004 | 0.986 | 1.023 | 0.659 |
|  |  | Inverse variance weighted | 0.004 | 0.007 | 1.004 | 0.990 | 1.018 | 0.623 |
|  |  | Weighted mode | 0.005 | 0.013 | 1.005 | 0.980 | 1.032 | 0.714 |
|  |  | Simple mode | 0.007 | 0.015 | 1.007 | 0.979 | 1.036 | 0.640 |
| DLBCL | 5alpha-pregnan-3beta,20alpha-diol monosulfate levels | MR Egger | 0.016 | 0.014 | 1.017 | 0.989 | 1.045 | 0.260 |
|  |  | Weighted median | 0.013 | 0.010 | 1.013 | 0.994 | 1.032 | 0.190 |
|  |  | Inverse variance weighted | 0.011 | 0.007 | 1.011 | 0.997 | 1.025 | 0.138 |
|  |  | Weighted mode | 0.023 | 0.018 | 1.023 | 0.988 | 1.060 | 0.220 |
|  |  | Simple mode | 0.020 | 0.018 | 1.020 | 0.985 | 1.056 | 0.289 |
| DLBCL | Androstenediol monosulfate levels | MR Egger | -0.024 | 0.012 | 0.976 | 0.953 | 0.999 | 0.065 |
|  |  | Weighted median | -0.006 | 0.009 | 0.994 | 0.976 | 1.012 | 0.506 |
|  |  | Inverse variance weighted | 0.005 | 0.007 | 1.005 | 0.991 | 1.020 | 0.464 |
|  |  | Weighted mode | -0.011 | 0.012 | 0.989 | 0.967 | 1.012 | 0.372 |
|  |  | Simple mode | -0.007 | 0.014 | 0.993 | 0.966 | 1.020 | 0.619 |
| DLBCL | 4-hydroxyglutamate levels | MR Egger | -0.015 | 0.015 | 0.986 | 0.958 | 1.014 | 0.336 |
|  |  | Weighted median | -0.007 | 0.010 | 0.993 | 0.974 | 1.013 | 0.519 |
|  |  | Inverse variance weighted | -0.008 | 0.007 | 0.992 | 0.978 | 1.007 | 0.298 |
|  |  | Weighted mode | -0.007 | 0.015 | 0.993 | 0.964 | 1.023 | 0.655 |
|  |  | Simple mode | 0.021 | 0.025 | 1.021 | 0.973 | 1.072 | 0.412 |
| DLBCL | N-formylphenylalanine levels | MR Egger | -0.001 | 0.015 | 0.999 | 0.971 | 1.029 | 0.956 |
|  |  | Weighted median | 0.011 | 0.011 | 1.011 | 0.990 | 1.033 | 0.308 |
|  |  | Inverse variance weighted | 0.011 | 0.008 | 1.011 | 0.996 | 1.026 | 0.163 |
|  |  | Weighted mode | 0.020 | 0.018 | 1.020 | 0.985 | 1.056 | 0.289 |
|  |  | Simple mode | 0.013 | 0.019 | 1.013 | 0.976 | 1.052 | 0.505 |
| DLBCL | Methyl-4-hydroxybenzoate sulfate levels | MR Egger | -0.006 | 0.013 | 0.994 | 0.968 | 1.021 | 0.669 |
|  |  | Weighted median | -0.007 | 0.010 | 0.993 | 0.974 | 1.011 | 0.432 |
|  |  | Inverse variance weighted | -0.005 | 0.007 | 0.995 | 0.982 | 1.009 | 0.469 |
|  |  | Weighted mode | -0.019 | 0.015 | 0.981 | 0.953 | 1.011 | 0.236 |
|  |  | Simple mode | 0.011 | 0.016 | 1.011 | 0.980 | 1.043 | 0.492 |
| DLBCL | Arabitol/xylitol levels | MR Egger | 0.019 | 0.014 | 1.019 | 0.991 | 1.047 | 0.211 |
|  |  | Weighted median | 0.006 | 0.009 | 1.006 | 0.988 | 1.024 | 0.526 |
|  |  | Inverse variance weighted | 0.002 | 0.007 | 1.002 | 0.987 | 1.016 | 0.827 |
|  |  | Weighted mode | 0.007 | 0.014 | 1.007 | 0.981 | 1.035 | 0.607 |
|  |  | Simple mode | 0.005 | 0.016 | 1.005 | 0.974 | 1.037 | 0.750 |
| DLBCL | Behenoyl dihydrosphingomyelin levels | MR Egger | -0.007 | 0.014 | 0.993 | 0.966 | 1.021 | 0.620 |
|  |  | Weighted median | -0.004 | 0.011 | 0.996 | 0.975 | 1.017 | 0.695 |
|  |  | Inverse variance weighted | -0.003 | 0.007 | 0.997 | 0.984 | 1.011 | 0.684 |
|  |  | Weighted mode | -0.008 | 0.014 | 0.992 | 0.966 | 1.019 | 0.584 |
|  |  | Simple mode | -0.010 | 0.016 | 0.990 | 0.959 | 1.022 | 0.549 |
| DLBCL | 1-myristoyl-2-arachidonoyl-GPC levels | MR Egger | -0.017 | 0.014 | 0.983 | 0.955 | 1.011 | 0.251 |
|  |  | Weighted median | -0.009 | 0.009 | 0.991 | 0.973 | 1.010 | 0.366 |
|  |  | Inverse variance weighted | -0.007 | 0.007 | 0.993 | 0.979 | 1.008 | 0.370 |
|  |  | Weighted mode | -0.004 | 0.014 | 0.996 | 0.969 | 1.024 | 0.800 |
|  |  | Simple mode | -0.007 | 0.017 | 0.993 | 0.960 | 1.027 | 0.699 |
| DLBCL | Glycosyl-N-tricosanoyl-sphingadienine levels | MR Egger | -0.016 | 0.015 | 0.984 | 0.955 | 1.015 | 0.327 |
|  |  | Weighted median | -0.002 | 0.011 | 0.998 | 0.977 | 1.020 | 0.891 |
|  |  | Inverse variance weighted | -0.001 | 0.008 | 0.999 | 0.984 | 1.015 | 0.898 |
|  |  | Weighted mode | -0.005 | 0.016 | 0.995 | 0.965 | 1.026 | 0.744 |
|  |  | Simple mode | -0.011 | 0.017 | 0.989 | 0.957 | 1.022 | 0.531 |
| DLBCL | Ceramide levels | MR Egger | 0.011 | 0.015 | 1.011 | 0.982 | 1.041 | 0.473 |
|  |  | Weighted median | 0.004 | 0.011 | 1.004 | 0.983 | 1.026 | 0.694 |
|  |  | Inverse variance weighted | 0.002 | 0.008 | 1.002 | 0.987 | 1.017 | 0.837 |
|  |  | Weighted mode | 0.007 | 0.015 | 1.007 | 0.978 | 1.037 | 0.651 |
|  |  | Simple mode | -0.001 | 0.019 | 0.999 | 0.963 | 1.037 | 0.973 |
| DLBCL | Dihomo-linolenoylcarnitine levels | MR Egger | 0.016 | 0.014 | 1.016 | 0.990 | 1.044 | 0.253 |
|  |  | Weighted median | 0.008 | 0.010 | 1.008 | 0.988 | 1.028 | 0.427 |
|  |  | Inverse variance weighted | 0.002 | 0.007 | 1.002 | 0.989 | 1.016 | 0.725 |
|  |  | Weighted mode | 0.010 | 0.014 | 1.010 | 0.983 | 1.038 | 0.485 |
|  |  | Simple mode | 0.005 | 0.018 | 1.005 | 0.969 | 1.042 | 0.793 |
| DLBCL | 8-methoxykynurenate levels | MR Egger | 0.031 | 0.015 | 1.031 | 1.001 | 1.062 | 0.061 |
|  |  | Weighted median | <0.001 | 0.011 | 0.999 | 0.978 | 1.023 | 0.996 |
|  |  | Inverse variance weighted | -0.006 | 0.009 | 0.994 | 0.976 | 1.011 | 0.478 |
|  |  | Weighted mode | 0.003 | 0.021 | 1.003 | 0.963 | 1.044 | 0.894 |
|  |  | Simple mode | -0.039 | 0.022 | 0.962 | 0.920 | 1.005 | 0.104 |
| DLBCL | 4-methylhexanoylglutamine levels | MR Egger | 0.028 | 0.020 | 1.028 | 0.989 | 1.069 | 0.180 |
|  |  | Weighted median | 0.024 | 0.012 | 1.024 | 1.000 | 1.049 | 0.050 |
|  |  | Inverse variance weighted | 0.005 | 0.010 | 1.005 | 0.985 | 1.026 | 0.605 |
|  |  | Weighted mode | 0.025 | 0.018 | 1.025 | 0.989 | 1.063 | 0.201 |
|  |  | Simple mode | 0.021 | 0.025 | 1.021 | 0.973 | 1.072 | 0.412 |
| DLBCL | 3-ureidopropionate levels | MR Egger | 0.010 | 0.014 | 1.010 | 0.982 | 1.039 | 0.491 |
|  |  | Weighted median | 0.007 | 0.010 | 1.007 | 0.988 | 1.027 | 0.471 |
|  |  | Inverse variance weighted | 0.015 | 0.007 | 1.015 | 1.001 | 1.030 | 0.030 |
|  |  | Weighted mode | 0.006 | 0.013 | 1.006 | 0.980 | 1.032 | 0.659 |
|  |  | Simple mode | 0.006 | 0.017 | 1.006 | 0.972 | 1.041 | 0.734 |
| DLBCL | Gamma-glutamylglutamine levels | MR Egger | 0.003 | 0.015 | 1.003 | 0.974 | 1.033 | 0.834 |
|  |  | Weighted median | -0.009 | 0.011 | 0.991 | 0.970 | 1.013 | 0.420 |
|  |  | Inverse variance weighted | -0.002 | 0.007 | 0.998 | 0.983 | 1.012 | 0.740 |
|  |  | Weighted mode | -0.025 | 0.021 | 0.975 | 0.936 | 1.015 | 0.233 |
|  |  | Simple mode | -0.022 | 0.023 | 0.978 | 0.935 | 1.023 | 0.351 |
| DLBCL | Citrate levels | MR Egger | 0.001 | 0.014 | 1.001 | 0.975 | 1.028 | 0.949 |
|  |  | Weighted median | 0.007 | 0.010 | 1.007 | 0.988 | 1.026 | 0.476 |
|  |  | Inverse variance weighted | 0.010 | 0.007 | 1.010 | 0.996 | 1.023 | 0.166 |
|  |  | Weighted mode | 0.003 | 0.015 | 1.003 | 0.974 | 1.032 | 0.857 |
|  |  | Simple mode | 0.011 | 0.016 | 1.011 | 0.980 | 1.044 | 0.490 |
| DLBCL | Cholesterol levels | MR Egger | -0.017 | 0.014 | 0.983 | 0.956 | 1.011 | 0.256 |
|  |  | Weighted median | -0.002 | 0.010 | 0.997 | 0.979 | 1.016 | 0.786 |
|  |  | Inverse variance weighted | -0.007 | 0.007 | 0.993 | 0.979 | 1.007 | 0.338 |
|  |  | Weighted mode | -0.001 | 0.013 | 0.999 | 0.975 | 1.025 | 0.976 |
|  |  | Simple mode | -0.001 | 0.015 | 0.999 | 0.969 | 1.029 | 0.931 |
| DLBCL | Androsterone sulfate levels | MR Egger | 0.011 | 0.013 | 1.011 | 0.985 | 1.038 | 0.411 |
|  |  | Weighted median | 0.007 | 0.010 | 1.007 | 0.989 | 1.026 | 0.439 |
|  |  | Inverse variance weighted | 0.010 | 0.007 | 1.010 | 0.997 | 1.024 | 0.138 |
|  |  | Weighted mode | 0.007 | 0.014 | 1.007 | 0.980 | 1.035 | 0.601 |
|  |  | Simple mode | 0.008 | 0.014 | 1.009 | 0.980 | 1.038 | 0.567 |

Abbreviation: MR,Mendelian randomization; SNP,single nucleotide polymorphisms; β, Beta; SE,standard error; OR, odds ratio; CI, confidence interval.

Supplement Table S6. Reverse Mendelian randomized analysis for the relation between metabolite ratios and Diffuse large B-cell lymphoma.

| **Exposure** | **Outcome** | **MR method** | **β** | **SE** | **OR** | **95% CI** | | ***P*-value** |
| --- | --- | --- | --- | --- | --- | --- | --- | --- |
| DLBCL | S-adenosylhomocysteine / 5-methyluridine | MR Egger | 0.007 | 0.016 | 1.007 | 0.977 | 1.039 | 0.643 |
|  |  | Weighted median | 0.003 | 0.011 | 1.003 | 0.982 | 1.025 | 0.781 |
|  |  | Inverse variance weighted | 0.001 | 0.008 | 1.000 | 0.985 | 1.016 | 0.978 |
|  |  | Weighted mode | 0.004 | 0.014 | 1.004 | 0.977 | 1.032 | 0.783 |
|  |  | Simple mode | -0.007 | 0.017 | 0.993 | 0.961 | 1.026 | 0.683 |
| DLBCL | Adenosine 5'-monophosphate / proline | MR Egger | -0.004 | 0.014 | 0.996 | 0.969 | 1.023 | 0.752 |
|  |  | Weighted median | 0.005 | 0.010 | 1.005 | 0.986 | 1.025 | 0.617 |
|  |  | Inverse variance weighted | -0.006 | 0.007 | 0.994 | 0.980 | 1.008 | 0.375 |
|  |  | Weighted mode | 0.009 | 0.015 | 1.009 | 0.981 | 1.039 | 0.538 |
|  |  | Simple mode | 0.009 | 0.019 | 1.009 | 0.973 | 1.047 | 0.637 |
| DLBCL | Serine / alpha-tocopherol | MR Egger | -0.018 | 0.014 | 0.983 | 0.956 | 1.010 | 0.224 |
|  |  | Weighted median | -0.015 | 0.010 | 0.985 | 0.966 | 1.004 | 0.113 |
|  |  | Inverse variance weighted | -0.005 | 0.007 | 0.995 | 0.981 | 1.009 | 0.489 |
|  |  | Weighted mode | -0.020 | 0.015 | 0.980 | 0.951 | 1.010 | 0.204 |
|  |  | Simple mode | 0.020 | 0.020 | 1.020 | 0.982 | 1.060 | 0.321 |
| DLBCL | Glutamate / glutamine | MR Egger | -0.005 | 0.014 | 0.995 | 0.969 | 1.022 | 0.719 |
|  |  | Weighted median | -0.001 | 0.010 | 0.999 | 0.981 | 1.018 | 0.958 |
|  |  | Inverse variance weighted | -0.002 | 0.007 | 0.998 | 0.985 | 1.011 | 0.733 |
|  |  | Weighted mode | 0.011 | 0.018 | 1.011 | 0.976 | 1.047 | 0.556 |
|  |  | Simple mode | 0.003 | 0.019 | 1.003 | 0.966 | 1.042 | 0.866 |
| DLBCL | Uridine / cytidine | MR Egger | 0.003 | 0.014 | 1.003 | 0.976 | 1.031 | 0.826 |
|  |  | Weighted median | 0.004 | 0.010 | 1.004 | 0.985 | 1.024 | 0.673 |
|  |  | Inverse variance weighted | 0.003 | 0.007 | 1.003 | 0.988 | 1.017 | 0.723 |
|  |  | Weighted mode | 0.002 | 0.014 | 1.002 | 0.975 | 1.031 | 0.865 |
|  |  | Simple mode | 0.001 | 0.017 | 1.001 | 0.969 | 1.034 | 0.951 |
| DLBCL | Adenosine 5'-diphosphate / glycerate | MR Egger | -0.007 | 0.019 | 0.993 | 0.957 | 1.030 | 0.725 |
|  |  | Weighted median | 0.006 | 0.013 | 1.006 | 0.981 | 1.031 | 0.652 |
|  |  | Inverse variance weighted | 0.005 | 0.010 | 1.005 | 0.986 | 1.024 | 0.622 |
|  |  | Weighted mode | 0.006 | 0.019 | 1.006 | 0.970 | 1.044 | 0.742 |
|  |  | Simple mode | 0.003 | 0.022 | 1.003 | 0.960 | 1.047 | 0.901 |
| DLBCL | Glycine / phosphate | MR Egger | -0.015 | 0.014 | 0.985 | 0.958 | 1.012 | 0.290 |
|  |  | Weighted median | -0.007 | 0.009 | 0.993 | 0.975 | 1.011 | 0.456 |
|  |  | Inverse variance weighted | -0.002 | 0.007 | 0.998 | 0.984 | 1.013 | 0.832 |
|  |  | Weighted mode | -0.009 | 0.014 | 0.991 | 0.965 | 1.018 | 0.524 |
|  |  | Simple mode | -0.006 | 0.015 | 0.994 | 0.965 | 1.024 | 0.705 |
| DLBCL | Cholate / bilirubin | MR Egger | -0.012 | 0.015 | 0.988 | 0.961 | 1.017 | 0.434 |
|  |  | Weighted median | 0.001 | 0.011 | 1.001 | 0.979 | 1.021 | 0.984 |
|  |  | Inverse variance weighted | -0.002 | 0.007 | 0.998 | 0.984 | 1.013 | 0.830 |
|  |  | Weighted mode | 0.007 | 0.017 | 1.007 | 0.974 | 1.041 | 0.694 |
|  |  | Simple mode | 0.016 | 0.021 | 1.016 | 0.975 | 1.059 | 0.459 |
| DLBCL | Cholate / adenosine 5'-monophosphate | MR Egger | -0.009 | 0.014 | 0.991 | 0.963 | 1.019 | 0.527 |
|  |  | Weighted median | 0.001 | 0.011 | 1.001 | 0.980 | 1.021 | 0.972 |
|  |  | Inverse variance weighted | -0.001 | 0.007 | 0.999 | 0.985 | 1.013 | 0.885 |
|  |  | Weighted mode | 0.002 | 0.017 | 1.002 | 0.970 | 1.035 | 0.916 |
|  |  | Simple mode | 0.004 | 0.019 | 1.004 | 0.968 | 1.041 | 0.845 |
| DLBCL | Taurine / glutamate | MR Egger | 0.009 | 0.013 | 1.009 | 0.984 | 1.035 | 0.491 |
|  |  | Weighted median | 0.002 | 0.009 | 1.002 | 0.984 | 1.021 | 0.810 |
|  |  | Inverse variance weighted | 0.001 | 0.007 | 1.001 | 0.987 | 1.013 | 0.987 |
|  |  | Weighted mode | 0.008 | 0.013 | 1.008 | 0.982 | 1.035 | 0.540 |
|  |  | Simple mode | 0.010 | 0.016 | 1.010 | 0.980 | 1.042 | 0.519 |
| DLBCL | Glutarate (C5-DC) / caprylate (8:0) | MR Egger | -0.002 | 0.018 | 0.998 | 0.963 | 1.033 | 0.898 |
|  |  | Weighted median | -0.003 | 0.011 | 0.997 | 0.976 | 1.019 | 0.782 |
|  |  | Inverse variance weighted | 0.003 | 0.009 | 1.003 | 0.986 | 1.021 | 0.722 |
|  |  | Weighted mode | -0.003 | 0.015 | 0.997 | 0.967 | 1.027 | 0.826 |
|  |  | Simple mode | -0.011 | 0.018 | 0.989 | 0.954 | 1.025 | 0.551 |
| DLBCL | Taurine / cysteine | MR Egger | 0.001 | 0.014 | 1.001 | 0.974 | 1.028 | 0.958 |
|  |  | Weighted median | 0.002 | 0.010 | 1.002 | 0.983 | 1.021 | 0.815 |
|  |  | Inverse variance weighted | -0.001 | 0.007 | 0.999 | 0.985 | 1.013 | 0.885 |
|  |  | Weighted mode | 0.002 | 0.013 | 1.002 | 0.976 | 1.028 | 0.903 |
|  |  | Simple mode | -0.001 | 0.015 | 0.999 | 0.971 | 1.029 | 0.981 |
| DLBCL | Phosphate / linoleoyl-arachidonoyl-glycerol (18:2-20:4) | MR Egger | 0.015 | 0.014 | 1.015 | 0.987 | 1.045 | 0.307 |
|  |  | Weighted median | 0.003 | 0.010 | 1.003 | 0.983 | 1.023 | 0.778 |
|  |  | Inverse variance weighted | 0.005 | 0.007 | 1.005 | 0.991 | 1.020 | 0.461 |
|  |  | Weighted mode | 0.001 | 0.016 | 1.001 | 0.971 | 1.032 | 0.933 |
|  |  | Simple mode | 0.001 | 0.016 | 1.001 | 0.970 | 1.032 | 0.957 |
| DLBCL | Tyrosine / pyruvate | MR Egger | -0.003 | 0.014 | 0.997 | 0.970 | 1.024 | 0.822 |
|  |  | Weighted median | -0.003 | 0.009 | 0.997 | 0.979 | 1.0160 | 0.785 |
|  |  | Inverse variance weighted | -0.005 | 0.007 | 0.995 | 0.982 | 1.009 | 0.521 |
|  |  | Weighted mode | -0.004 | 0.014 | 0.996 | 0.969 | 1.024 | 0.795 |
|  |  | Simple mode | -0.001 | 0.015 | 0.999 | 0.970 | 1.029 | 0.953 |
| DLBCL | Succinate / proline | MR Egger | 0.003 | 0.014 | 1.004 | 0.977 | 1.031 | 0.802 |
|  |  | Weighted median | 0.003 | 0.010 | 1.003 | 0.984 | 1.022 | 0.774 |
|  |  | Inverse variance weighted | 0.008 | 0.007 | 1.008 | 0.994 | 1.022 | 0.254 |
|  |  | Weighted mode | 0.006 | 0.014 | 1.006 | 0.979 | 1.034 | 0.691 |
|  |  | Simple mode | 0.003 | 0.016 | 1.003 | 0.971 | 1.035 | 0.867 |
| DLBCL | Phosphate / EDTA | MR Egger | 0.027 | 0.013 | 1.027 | 1.001 | 1.054 | 0.063 |
|  |  | Weighted median | 0.005 | 0.009 | 1.005 | 0.987 | 1.024 | 0.563 |
|  |  | Inverse variance weighted | -0.002 | 0.007 | 0.998 | 0.985 | 1.012 | 0.799 |
|  |  | Weighted mode | 0.014 | 0.014 | 1.014 | 0.987 | 1.042 | 0.322 |
|  |  | Simple mode | 0.008 | 0.018 | 1.008 | 0.973 | 1.043 | 0.677 |
| DLBCL | Adenosine 5'-diphosphate / mannitol to sorbitol | MR Egger | -0.006 | 0.019 | 0.994 | 0.958 | 1.031 | 0.746 |
|  |  | Weighted median | 0.012 | 0.013 | 1.012 | 0.986 | 1.039 | 0.360 |
|  |  | Inverse variance weighted | 0.014 | 0.010 | 1.014 | 0.995 | 1.033 | 0.147 |
|  |  | Weighted mode | 0.006 | 0.019 | 1.006 | 0.970 | 1.043 | 0.747 |
|  |  | Simple mode | 0.001 | 0.022 | 1.001 | 0.960 | 1.045 | 0.946 |
| DLBCL | Phosphoethanolamine / choline | MR Egger | 0.007 | 0.014 | 1.007 | 0.980 | 1.035 | 0.629 |
|  |  | Weighted median | 0.001 | 0.010 | 1.001 | 0.981 | 1.022 | 0.902 |
|  |  | Inverse variance weighted | 0.001 | 0.007 | 1.001 | 0.987 | 1.014 | 0.936 |
|  |  | Weighted mode | 0.002 | 0.014 | 1.002 | 0.975 | 1.029 | 0.908 |
|  |  | Simple mode | 0.002 | 0.017 | 1.001 | 0.969 | 1.035 | 0.925 |
| DLBCL | Serine / threonine | MR Egger | 0.002 | 0.014 | 1.002 | 0.975 | 1.029 | 0.899 |
|  |  | Weighted median | 0.013 | 0.010 | 1.013 | 0.994 | 1.032 | 0.183 |
|  |  | Inverse variance weighted | 0.011 | 0.007 | 1.011 | 0.997 | 1.025 | 0.115 |
|  |  | Weighted mode | 0.012 | 0.013 | 1.013 | 0.986 | 1.040 | 0.371 |
|  |  | Simple mode | 0.012 | 0.016 | 1.012 | 0.981 | 1.043 | 0.458 |

Abbreviation: MR,Mendelian randomization; SNP,single nucleotide polymorphisms; β, Beta; SE,standard error; OR, odds ratio; CI, confidence interval.

Supplement Table S7. Reverse Mendelian randomized heterogeneity test and pleiotropy test of Diffuse large B-cell lymphoma and gut microbiota, plasma metabolites, and metabolite ratios .

| **Exposure** | **Outcome** | **MR-Egger-intercept**  ***P-*value** | **Cochran’s Q**  ***P-*value** |
| --- | --- | --- | --- |
| DLBCL | Terrisporobacter | 0.506 | 0.914(MR Egger) |
|  |  |  | 0.926(IVW) |
| DLBCL | Methanobrevibacter | 0.942 | 0.396(MR Egger) |
|  |  |  | 0.495(IVW) |
| DLBCL | Eubacterium coprostanoligenes group | 0.765 | 0.374(MR Egger) |
|  |  |  | 0.456(IVW) |
| DLBCL | Slackia | 0.577 | 0.645(MR Egger) |
|  |  |  | 0.701(IVW) |
| DLBCL | Oscillibacter | 0.692 | 0.796(MR Egger) |
|  |  |  | 0.849(IVW) |
| DLBCL | Methionine sulfoxide levels | 0.924 | 0.913(MR Egger) |
|  |  |  | 0.942(IVW) |
| DLBCL | DHEAS levels | 0.017 | 0.779(MR Egger) |
|  |  |  | 0.311(IVW) |
| DLBCL | 3-methyl-2-oxobutyrate levels | 0.442 | 0.413(MR Egger) |
|  |  |  | 0.441(IVW) |
| DLBCL | 2,3-dihydroxypyridine levels | 0.431 | 0.567(MR Egger) |
|  |  |  | 0.591(IVW) |
| DLBCL | Glycolithocholate levels | 0.994 | 0.820(MR Egger) |
|  |  |  | 0.868(IVW) |
| DLBCL | 5-dodecenoate levels | 0.744 | 0.905(MR Egger) |
|  |  |  | 0.931(IVW) |
| DLBCL | Alpha-hydroxyisovalerate levels | 0.241 | 0.605(MR Egger) |
|  |  |  | 0.563(IVW) |
| DLBCL | N-methyl-2-pyridone-5-carboxamide levels | 0.009 | 0.640(MR Egger) |
|  |  |  | 0.145(IVW) |
| DLBCL | 4-ethylphenylsulfate levels | 0.647 | 0.984(MR Egger) |
|  |  |  | 0.989(IVW) |
| DLBCL | 5alpha-pregnan-3beta,20alpha-diol monosulfate levels | 0.635 | 0.689(MR Egger) |
|  |  |  | 0.738(IVW) |
| DLBCL | Androstenediol monosulfate levels | 0.013 | 0.621(MR Egger) |
|  |  |  | 0.178(IVW) |
| DLBCL | 4-hydroxyglutamate levels | 0.596 | 0.686(MR Egger) |
|  |  |  | 0.732(IVW) |
| DLBCL | N-formylphenylalanine levels | 0.385 | 0.614(MR Egger) |
|  |  |  | 0.625(IVW) |
| DLBCL | Methyl-4-hydroxybenzoate sulfate levels | 0.939 | 0.837(MR Egger) |
|  |  |  | 0.882(IVW) |
| DLBCL | Arabitol/xylitol levels | 0.185 | 0.278(MR Egger) |
|  |  |  | 0.218(IVW) |
| DLBCL | Behenoyl dihydrosphingomyelin levels | 0.728 | 0.372(MR Egger) |
|  |  |  | 0.435(IVW) |
| DLBCL | 1-myristoyl-2-arachidonoyl-GPC levels | 0.401 | 0.307(MR Egger) |
|  |  |  | 0.321(IVW) |
| DLBCL | Glycosyl-N-tricosanoyl-sphingadienine levels | 0.288 | 0.321(MR Egger) |
|  |  |  | 0.304(IVW) |
| DLBCL | Ceramide levels | 0.476 | 0.697(MR Egger) |
|  |  |  | 0.724(IVW) |
| DLBCL | Dihomo-linolenoylcarnitine levels | 0.258 | 0.730(MR Egger) |
|  |  |  | 0.692(IVW) |
| DLBCL | 8-methoxykynurenate levels | 0.013 | 0.611(MR Egger) |
|  |  |  | 0.166(IVW) |
| DLBCL | 4-methylhexanoylglutamine levels | 0.205 | 0.126(MR Egger) |
|  |  |  | 0.091(IVW) |
| DLBCL | 3-ureidopropionate levels | 0.675 | 0.377(MR Egger) |
|  |  |  | 0.436(IVW) |
| DLBCL | Gamma-glutamylglutamine levels | 0.667 | 0.299(MR Egger) |
|  |  |  | 0.352(IVW) |
| DLBCL | Citrate levels | 0.468 | 0.647(MR Egger) |
|  |  |  | 0.675(IVW) |
| DLBCL | Cholesterol levels | 0.429 | 0.333(MR Egger) |
|  |  |  | 0.354(IVW) |
| DLBCL | Androsterone sulfate levels | 0.918 | 0.795(MR Egger) |
|  |  |  | 0.847(IVW) |
| DLBCL | S-adenosylhomocysteine to 5-methyluridine ratio | 0.600 | 0.402(MR Egger) |
|  |  |  | 0.455(IVW) |
| DLBCL | Adenosine 5'-monophosphate to proline ratio | 0.882 | 0.727(MR Egger) |
|  |  |  | 0.788(IVW) |
| DLBCL | Serine to alpha-tocopherol ratio | 0.303 | 0.834(MR Egger) |
|  |  |  | 0.814(IVW) |
| DLBCL | Glutamate to glutamine ratio | 0.821 | 0.382(MR Egger) |
|  |  |  | 0.451(IVW) |
| DLBCL | Uridine to cytidine ratio | 0.961 | 0.836(MR Egger) |
|  |  |  | 0.881(IVW) |
| DLBCL | Adenosine 5'-diphosphate to glycerate ratio | 0.488 | 0.931(MR Egger) |
|  |  |  | 0.939(IVW) |
| DLBCL | Glycine to phosphate ratio | 0.269 | 0.445(MR Egger) |
|  |  |  | 0.423(IVW) |
| DLBCL | Cholate to bilirubin ratio | 0.432 | 0.472(MR Egger) |
|  |  |  | 0.498(IVW) |
| DLBCL | Cholate to adenosine 5'-monophosphate ratio | 0.515 | 0.742(MR Egger) |
|  |  |  | 0.772(IVW) |
| DLBCL | Taurine to glutamate ratio | 0.430 | 0.738(MR Egger) |
|  |  |  | 0.754(IVW) |
| DLBCL | Glutarate to caprylate ratio | 0.727 | 0.078(MR Egger) |
|  |  |  | 0.102(IVW) |
| DLBCL | Taurine to cysteine ratio | 0.884 | 0.626(MR Egger) |
|  |  |  | 0.696(IVW) |
| DLBCL | Phosphate to linoleoyl-arachidonoyl-glycerol ratio | 0.440 | 0.983(MR Egger) |
|  |  |  | 0.982(IVW) |
| DLBCL | Tyrosine to pyruvate ratio | 0.910 | 0.943(MR Egger) |
|  |  |  | 0.963(IVW) |
| DLBCL | Succinate to proline ratio | 0.708 | 0.519(MR Egger) |
|  |  |  | 0.583(IVW) |
| DLBCL | Phosphate to EDTA ratio | 0.025 | 0.767(MR Egger) |
|  |  |  | 0.370(IVW) |
| DLBCL | Adenosine 5'-diphosphate to mannitol to sorbitol ratio | 0.232 | 0.725(MR Egger) |
|  |  |  | 0.674(IVW) |
| DLBCL | Phosphoethanolamine to choline ratio | 0.606 | 0.391(MR Egger) |
|  |  |  | 0.444(IVW) |
| DLBCL | Serine to threonine ratio | 0.303 | 0.818(MR Egger) |
|  |  |  | 0.831(IVW) |
